# Four new acylated glycosidic acid methyl esters and a new glycosidic acid from *Ipomoea lacunosa* seeds

**DOI:** 10.1007/s11418-025-01877-8

**Published:** 2025-02-13

**Authors:** Masateru Ono, Renjyu Murakami, Shin Yasuda, Hiroyuki Miyashita, Hitoshi Yoshimitsu, Ryota Tsuchihashi, Masafumi Okawa, Junei Kinjo

**Affiliations:** 1 School of Agriculture, Tokai University, 871-12 Sugido, Mashiki-Cho, Kamimashiki-Gun, Kumamoto, 861-2205 Japan; 2 Faculty of Pharmaceutical Sciences, Sojo University, 4-22-1 Ikeda, Nishi-Ku, Kumamoto, 860-0082 Japan; 3 Faculty of Pharmaceutical Sciences, Fukuoka University, 8-19-1 Nanakuma, Jonan-Ku, Fukuoka, 814-0180 Japan

**Keywords:** Resin glycoside, *Ipomoea lacunosa*, Convolvulaceae, Acylated glycosidic acid methyl ester, Glycosidic acid, 4,11-Dihydroxyhexadecanoic acid

## Abstract

Resin glycosides, characteristic of plants of the Convolvulaceae family, are well-known purgative constituents found in traditional medicinal crude drugs, such as Rhizoma Jalapae, Rhizoma Jalapae Braziliensis, Orizaba Jalapa Tuber, and Pharbitidis Semen. The isolated compounds exhibited a wide range of biological activities, including antibacterial, ionophoric, anti-inflammatory, antiviral, and multidrug-resistance-modulating properties, as well as cytotoxicity against cancer cells. *Ipomoea lacunosa* L. (Convolvulaceae) is an herbaceous vine native to the United States. Four new acylated glycosidic acid methyl esters (**1**–**4**) and one new glycosidic acid (**5**) were isolated from the methanol extract of the plant seed. The structures of **1**–**5** were elucidated using spectroscopic data in conjunction with our previous studies on the components of the crude resin glycoside fraction from *I. lacunosa* seeds. Compounds **1** and **2** were identified as heptaglycosides, **3** as an octaglycoside, and **4** as a nonaglycoside, all sharing methyl 3*S*,11*S*-dihydroxytetradecanoate as a common aglycone. The saccharide moieties were partially acylated by glycosidic acid, 7*S*-hydroxydecanoic acid 7-*O*-*β*-d-quinovopyranoside, and organic acids, including (*E*)-2-methylbut-2-enoic, 2*S*-methylbutyric, and 2*R*-methyl-3*R*-hydroxybutyric acids. Compound **5** was identified as a triglycoside with a new aglycone, 4,11-dihydroxyhexadecanoic acid, which is the first glycosidic acid with a hydroxyl group at C-4 of the aglycone moiety.

**Graphical abstract:**

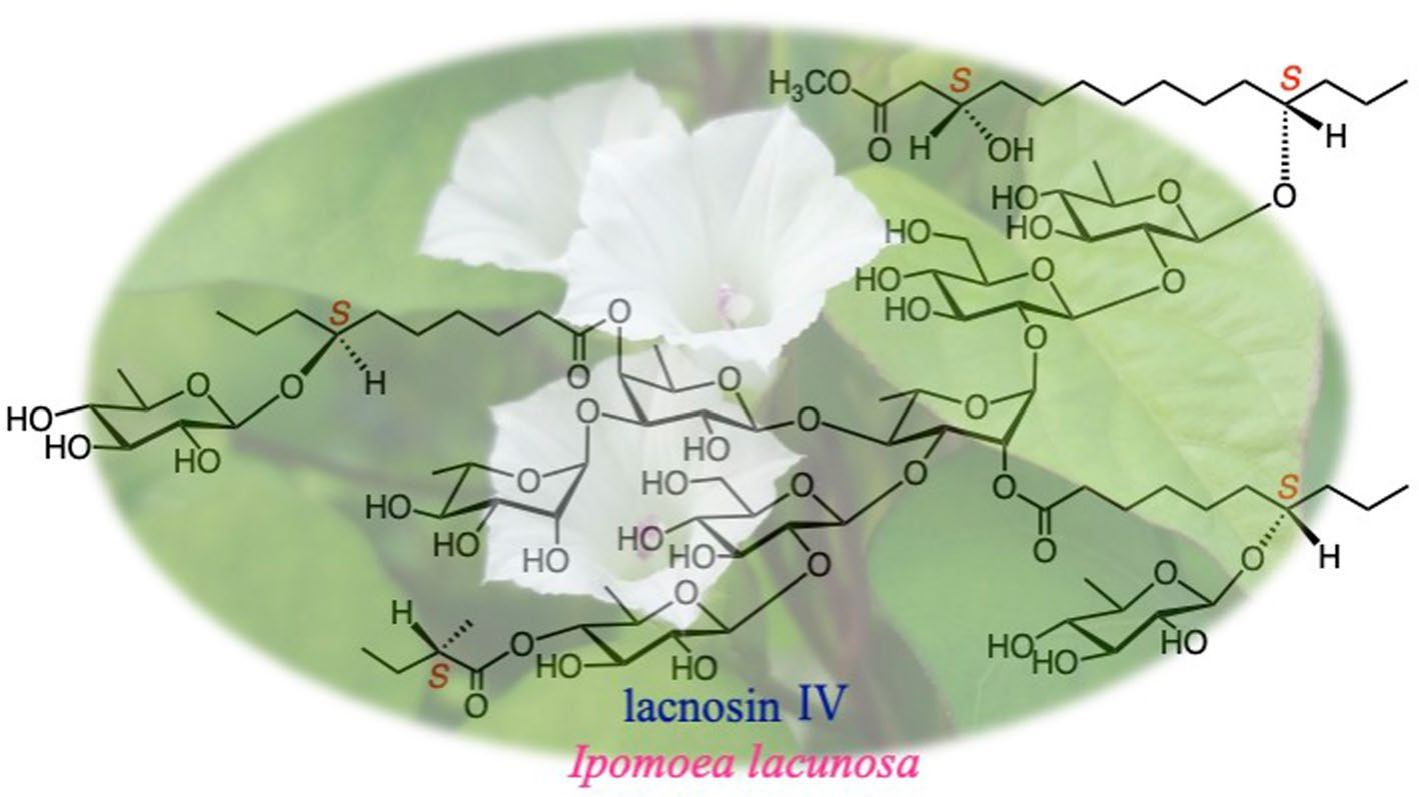

**Supplementary Information:**

The online version contains supplementary material available at 10.1007/s11418-025-01877-8.

## Introduction

Resin glycosides are well-known purgative ingredients that are characteristic of some crude drugs, such as Rhizoma Jalapae (the root of *I. purga* (Wender) Hayne), Rhizoma Jalapae Braziliensis (the root of *I. operculata* (Gomes) Mart.), Orizaba Jalap Tuber (the tuber of *Ipomoea orizabensis* (Pelletan) Leded.), and Pharbitidis Semen (the seed of *Ipomoea nil* Choisy), which are commonly found in plants belonging to the Convolvulaceae family [1, 2]. Chemically, resin glycosides have a core structure in which the sugar moiety of the oligoglycoside linked to a hydroxyl fatty acid (glycosidic acid) is partially acylated by several organic acids [1, 2]. They are broadly classified as jalapins, which feature an intramolecular cyclic structure, and convolvulins, which have a free carboxyl group on their aglycones [1]. Some biological effects of resin glycosides have been reported, such as cytotoxicity against cancer cells [2–4] and antibacterial [2], ionophoric [2], anti-inflammatory [5], drug-resistance-modulatory [6], and antiviral [3, 7] activities.

*Ipomoea lacunosa* L. (Convolvulaceae) is an herbaceous vine native to the United States [8]. Regarding the resin glycosides of this plant, the glycosidic acid component of the resin glycoside fraction extracted from the roots of this plant has been proposed to be a heptaglycoside of 11-hydroxyhexadecanoic (jalapinolic) acid with a glucose-to-rhamnose molar ratio of approximately 2:5; however, its detailed structure has not yet been determined [9]. In a previous study, we reported four monosaccharides (d-glucose, d-fucose, d-quinovose, and l-rhamnose), three organic acids [(*E*)-2-methylbut-2-enoic (tiglic), 2*S*-methylbutyric, and 2*R*-methyl-3*R*-hydroxybutyric (2*R*,3*R*-nilic) acids], and six hydroxyl fatty acids [7*S*-hydroxydecanoic, 11*S*-hydroxytetradecanoic, 11*S*-jalapinolic, 3*S*,11*S*-dihydroxytetradecanoic (ipurolic), 3*S*,11*S*-dihydroxyhexadecanoic, and 3*S*,12*S*-dihydroxyhexadecanoic acids] as the components of the crude resin glycoside fraction of *I. lacunosa* seeds [10]. The isolation and structural elucidation of 11 new glycosidic acid methyl esters (lacunosinic acids A–H methyl esters and multifidinic acids C, D, and F methyl esters) and two known glycosidic acid methyl esters (quamoclinic acid B methyl ester and purgic acid A methyl ester) are also reported [10]. Continuing our previous studies on resin glycosides from Convolvulaceae plants [11], this report describes the isolation and structural elucidation of four new acylated glycosidic acid methyl esters and a new glycosidic acid from *I. lacunosa* seeds.

## Results and discussion

The methanol (MeOH) extract of *I. lacunosa* seeds was partitioned between hexane and 95% MeOH. The 95% MeOH-soluble fr. was chromatographed on Diaion HP20, Sephadex LH-20, and octadecyl silica (ODS) columns, and HPLC using ODS furnished five compounds (**1**–**5**) as amorphous powders.

**Fig. 1:**
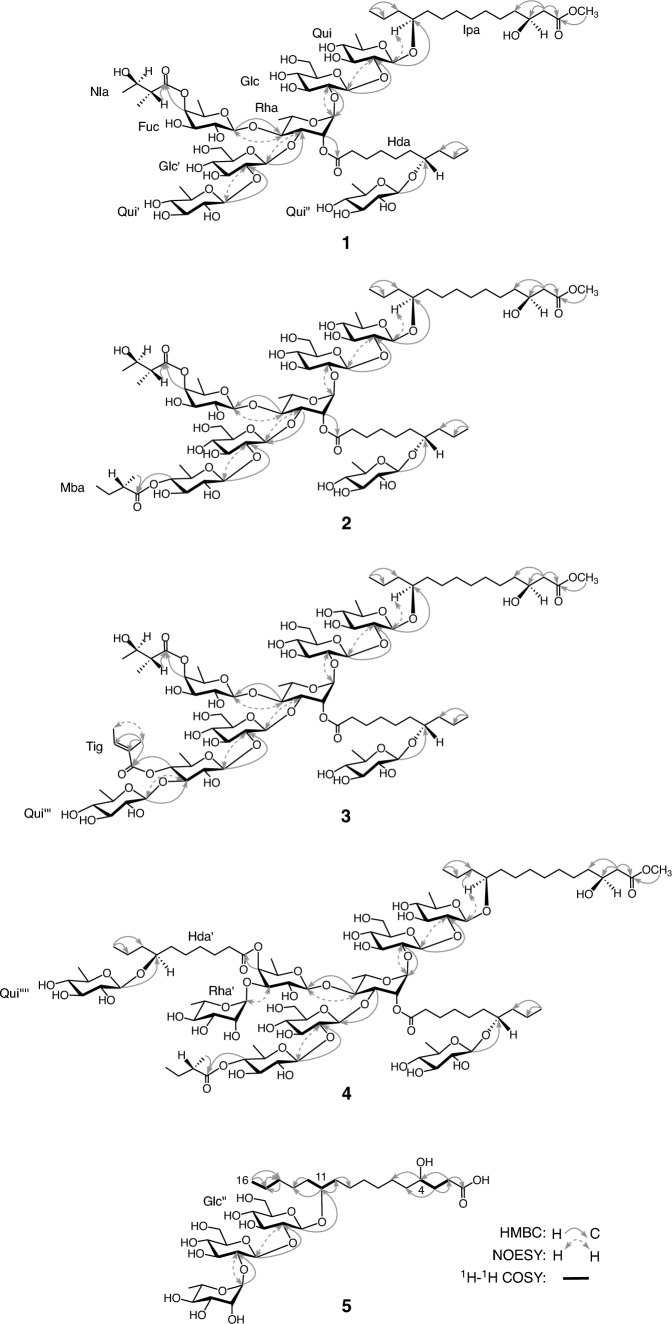
Key HMBC and NOESY correlations observed for **1–5** and key ^1^H-^1^H COSY correlations observed for **5** (600 MHz, in pyridine-*d*_5_)

**Fig. 2:**
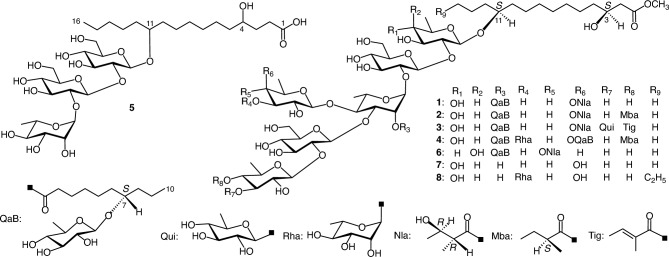
Structures of **1**–**8**

The HR-positive-ion and HR-negative-ion electrospray ionization time-of-flight mass spectrometry (ESI-TOF-MS) data for **1** (lacunosin I) showed [M + Na]^+^ and [M–H]^–^ ion peaks at *m/z* 1621.7816 and 1597.7852, respectively, indicating a molecular formula of C_72_H_126_O_38_. The ^1^H-NMR spectrum of **1** revealed signals due to seven anomeric protons [*δ* 6.47 (1H, d, *J* = 1.5 Hz), 6.36 (1H, d, *J* = 7.5 Hz), 5.96 (1H, d, *J* = 8.0 Hz), 5.84 (1H, d. *J* = 7.5 Hz), 5.12 (1H, d, *J* = 7.5 Hz), 4.87 (1H, d, *J* = 7.5 Hz), 4.80 (1H, d, *J* = 7.5 Hz)], one methoxy group [*δ* 3.64 (3H, s)], two sets of non-equivalent methylene protons [*δ* 2.73 (1H, dd, *J* = 7.5, 15.5 Hz), 2.71 (1H, dd, *J* = 5.0, 15.5 Hz); *δ* 2.62 (1H, ddd, *J* = 8.0, 9.0, 15.5 Hz), 2.51 (1H, ddd, *J* = 7.0, 9.0, 15.5 Hz)] adjacent to the carbonyl groups, seven secondary methyl groups [*δ* 1.97 (3H, d, *J* = 6.0 Hz), 1.83 (3H, d, *J* = 6.0 Hz), 1.63 (3H, d, *J* = 6.0 Hz), 1.60 (3H, d, *J* = 6.0 Hz), 1.47 (3H, d, *J* = 6.0 Hz), 1.45 (3H, d, *J* = 7.5 Hz), 1.35 (3H, d, *J* = 7.5 Hz)], and two primary methyl groups [*δ* 0.97 (3H, t, *J* = 7.0 Hz), 0.93 (3H, t, *J* = 7.0 Hz)], together with a signal presumably due to one H-2 [*δ* 2.81 (1H, dq, *J* = 7.5, 7.5 Hz)] of the niloyl residue. The ^13^C-NMR spectrum shows signals corresponding to three carboxyl (*δ* 174.8, 173.7, 172.9) and seven anomeric carbons (*δ* 106.2, 103.5, 102.7, 102.1, 101.7, 101.2, 96.8). The ^1^H- and ^13^C-NMR signals were assigned based on the ^1^H-^1^H COSY, ^1^H-^1^H total correlation spectroscopy (TOCSY), HMQC, HMBC, and NOESY spectra (Tables 1 and 2). The coupling constant of the ^1^H-NMR signals attributed to the anomeric and methine protons, along with the C–H coupling constant (^1^*J*_C-1–H-1_) of the anomeric carbon signals [12] observed in the ^13^C-NMR spectrum, indicated that the monosaccharide units of **1** consisted of 1 mol each of fucopyranose with *β* in ^4^C_1_ conformation and rhamnopyranose with *α* in ^1^C_4_ conformation, 2 mol of glucopyranose with *β* in ^4^C_1_ conformation, and 3 mol of quinovopyranose with *β* in ^4^C_1_ conformation. The absolute configurations of the monosaccharide components quinovose, glucose, fucose, and rhamnose of the crude resin glycoside fraction of *I. lacunosa* seeds were previously identified as d, d, d, and l, respectively, based on the HPLC analysis of their thiocarbamoyl-thiazolidine derivatives [10]. In contrast, the organic acid component of **1** was identified as 1 mol of nilic acid. The absolute configuration of the nilic acid component of the crude resin glycoside fraction of *I. lacunosa* seeds was previously confirmed to be 2*R*,3*R* through specific rotation measurement and HPLC analyses with a chiral stationary phase of its *p*-bromophenacyl ester [10]. In the HMBC spectrum of **1**, important correlations are observed between the signals of H-1 of the first glucosyl residue (Glc) and C-2 of the first quinovosyl residue (Qui); H-2 of Glc and C-1 of the first rhamnosyl residue (Rha); H-1 of the second glucosyl residue (Glc') and C-3 of Rha; H-1 of the fucosyl residue (Fuc) and C-4 of Rha; and H-1 of the second quinovosyl residue (Qui') and C-2 of Glc' (Fig. 1). Furthermore, key cross-peaks in the NOESY spectrum of **1** are detected between the signals of H-1 Glc and H-2 of Qui; H-1 of Rha and H-2 of Glc; H-1 of Glc' and H-3 of Rha; H-1 of Fuc and H-4 of Rha; and H-1 of Qui' and H-2 of Glc' (Fig. 1). Thus, **1** is composed of the sugar chain of *β*-d-quinovopyranosyl-(1 → 2)-*O*-*β*-d-glucopyranosyl-(1 → 3)-[*O*-*β*-d-fucopyranosyl-(1 → 4)]-*O*-*α*-l-rhamnopyranosyl-(1 → 2)-*O*-*β*-d-glucopyranosyl-(1 → 2)-*β*-D-quinovopyranose. In a previous study, we demonstrated that the chemical shift of the methylene carbon (*β*-carbon) at the β-position of the terminal methyl group of the aglycone moiety (Agl) in the resin glycoside, as observed in the ^13^C-NMR spectrum, served as a reliable indicator of the position of the hydroxyl group of the aglycone [13]. Specifically, the chemical shift of the *β*-carbon varied significantly depending on the number of methylene groups between the oxymethine and terminal methyl groups of Agl. The chemical shift at the *β*-carbon in pyridine-*d*_5_ was approximately *δ*_c_ 32.5 when there were four methylene groups, approximately *δ*_c_ 27.5 when there were three methylene groups, and approximately *δ*_c_ 37.5 when there were two methylene groups. Based on the chemical shift (*δ* 37.7) of the *β*-carbon of the terminal methyl group observed in the ^13^C-NMR spectrum, the ^1^H-^1^H COSY correlations between the signals of H_2_-2 (*δ* 2.73, 2.71) of Agl and H-3 (*δ* 4.42) of Agl, the HMBC correlation between the signals of the methoxy protons and C-1 (*δ* 172.9) of Agl; the aglycone of **1** was identified as methyl ipurolate. The absolute configuration of the ipurolic acid component in the crude resin glycoside fraction of *I. lacunosa* seeds is the 3*S*,11*S*, which was previously determined from the ^1^H-NMR spectrum of the (+)-*α*-methoxy-*α*-trifluoromethylphenylacetic acid (MTPA) derivative of its methyl ester [10]. In addition, glycosylation shifts [14, 15] at C-10 (–3.8 or –3.9 ppm), C-11 (+ 10.2 ppm), and C-12 (–3.0 ppm) of the methyl ipurolate moiety (Ipa) were detected compared with the ^13^C-NMR data for methyl ipurolate in the literature [16]. A correlation in the HMBC spectrum of **1** was observed between the signals of H-1 of Qui and C-11 of Ipa. These data suggest that **1** is composed of multifidinic acid C [17], which was previously reported to be the glycosidic acid component of the crude resin glycoside fraction from *I. lacunosa* seeds [10]. Furthermore, from the unassigned NMR signals corresponding to one primary methyl group, a *β*-d-quinovopyranosyl residue, an oxymethine carbon, a *β*-carbon (*δ* 37.4), and a carboxyl carbon, in conjunction with the molecular formula of **1**, it was suggested that **1** was composed of quamoclinic acid B [18], a glycosidic acid component of the crude resin glycoside fraction of *I. lacunosa* seeds [10]. The absolute configuration of the aglycone in quamoclinic acid B (7-hydroxydecanoic acid) was previously determined through ^1^H-NMR analysis of the (+)-MTPA derivative of its methyl ester, which was obtained during our studies on the components of the crude resin glycoside fraction [10]. The presence of quamoclinic acid B as a component of **1** is supported by the following evidence. A correlation was observed in the HMBC spectrum of **1** between the signals of H-1 of the third quinovosyl residue (Qui'') and C-7 of the first 7*S*-hydroxydecanoyl residue [Hda; Agl of the first quamoclinic acid B residue]. The negative-ion ESI-TOF-MS/MS of [M–H]^–^ ion showed a fragment ion peak at *m/z* 1281.5956 [M–(quamoclinic acid B unit)]^–^. Based on these findings, it can be inferred that **1** has a structure analogous to that of QM-11 (**6**) [19]. Compound **6** is composed of 1 mol each of 2*R*,3*R*-nilic acid and quamoclinic acid B, which are linked via ester bonds to the sugar moiety of the methyl ester of quamoclinic acid C [18]. Quamoclinic acid C is an epimer of multifidinic acid C, in which the Qui and Fuc are substituted with *β*-D-fucopyranosyl and *β*-D-quinovopyranosyl residues, respectively (Fig. 2). Comparison of the ^1^H-NMR spectra of **1** and multifidinic acid C methyl ester (**7**) [10] indicated remarkable downfield shifts (∆*δ* = *δ***1**–*δ***7**) of the signals corresponding to H-2 (∆*δ* = 1.10) of Rha and H-4 (∆*δ* = 1.65) of Fuc due to acylation. These data suggested that the ester linkages were located at C-2 of Rha and C-4 of Fuc. In addition, the HMBC spectrum of **1** showed key cross-peaks between the signals of H-2 of Rha and C-1 of Hda, and H-4 of Qui and C-1 of the niloyl residue (Nla) (Fig. 1). Accordingly, the structure of **1** was defined as methyl 3*S*,11*S*-ipurolate 11-*O*-*β*-d-quinovopyranosyl-(1 → 2)-*O*-*β*-d-glucopyranosyl-(1 → 3)-[*O*-(4-*O*-2*R*,3*R*-niloyl)-*β*-d-fucopyranosyl-(1 → 4)]-*O*-(2-*O*-7*S*-hydroxydecanoyl 7-*O*-*β*-d-quinovopyranoside)-*α*-l-rhamnopyranosyl-(1 → 2)-*O*-*β*-d-glucopyranosyl-(1 → 2)-*β*-d-quinovopyranoside, which is an isomer of **6**, in which the glycosidic acid component of **6** was substituted by multifidinic acid C (Fig. 2). The NMR assignments of Ipa and Hda were performed with according to previously reported data [10, 20].

**Table 1:** ^1^H-NMR spectroscopic data for **1**–**4** (600 MHz, in pyridine-*d*_5_)

	**1**	**2**	**3**	**4**
Qui-1	4.87 d(7.5)	4.88 d(7.5)	4.88 d(8.0)	4.81 d(7.5)
2	4.23 dd(7.5, 9.0)	4.23 dd(7.5, 9.0)	4.23^a^	4.18^a^
3	4.76 dd(9.0, 9.0)	4.79^a^	4.80 dd(9.0, 9.0)	4.67^a^
4	3.55 dd(9.0, 9.0)	3.55 dd(9.0, 9.0)	3.56 dd(9.0, 9.0)	3.58 dd(9.0, 9.0)
5	3.92 dd(9.0, 6.0)	3.93^a^	3.95 dq(9.0, 6.0)	3.85 dq(9.0, 6.0)
6	1.60 d(6.0)	1.60 d(5.5)	1.61 d(6.0)	1.55 d(6.0)
Glc-1	5.84 d(7.5)	5.85 d(7.5)	5.86 d(8.0)	5.79 d(7.5)
2	4.26 dd(7.5, 9.0)	4.27 dd(7.5, 8.5)	4.25^a^	4.24^a^
3	4.04^a^	4.04^a^	4.05^a^	4.06^a^
4	3.98^a^	4.00^a^	3.99^a^	4.00^a^
5	3.69 ddd(3.5, 5.5, 9.0)	3.68 ddd(3.5, 7.0, 9.0)	3.67 ddd(3.0, 6.0, 9.0)	3.69 ddd(3.5, 6.0, 9.0)
6	4.41^a^	4.39^a^	4.40^a^	4.39^a^
6	4.23^a^	4.22^a^	4.22^a^	4.20^a^
Rha-1	6.47 d(1.5)	6.48 s	6.47 br s	6.45 s
2	5.95 dd(1.5, 3.5)	5.92 d(3.0)	5.92 d(3.5)	5.94 d(3.0)
3	5.49 dd(3.5, 9.0)	5.51 dd(3.0, 9.5)	5.48 dd(3.5, 9.5)	5.46 dd(3.0, 9.0)
4	4.70 dd(9.0, 9.0)	4.65 dd(9.5, 9.5)	4.62 dd(9.5, 9.5)	4.65 dd(9.0, 9.0)
5	5.29 dd(9.0, 6.0)	5.29 dq(9.5, 6.0)	5.27 dq(9.6, 6.0)	5.19 dq(9.0, 6.0)
6	1.97 d(6.0)	1.97 d(6.0)	1.94 d(6.0)	1.94 d(6.0)
Glc'-1	6.36 d(7.5)	6.43 d(8.0)	6.41 d(7.5)	6.23 d(7.5)
2	3.99 dd(7.5, 9.0)	4.00^a^	3.91 dd(7.5, 9.0)	3.97^a^
3	4.51 dd(9.0, 9.0)	4.52 dd(9.0, 9.0)	4.48 dd(9.0, 9.0)	4.43 dd(9.0, 9.0)
4	3.86^a^	3.83 dd(9.0, 9.0)	3.80^a^	3.78^a^
5	4.33^a^	4.36^a^	4.35^a^	4.19^a^
6	4.53^a^	4.53^a^	4.52 br d(11.0)	4.45^a^
6	4.08^a^	4.06^a^	4.06^a^	4.03^a^
Fuc-1	5.96 d(8.0)	5.93 d(8.0)	5.90 d(7.5)	5.90 d(7.5)
2	4.22^a^	4.18 dd(8.0, 9.0)	4.18 dd(7.5, 9.0)	4.31^a^
3	4.37^a^	4.32 dd(3.0, 9.0)	4.31 dd(3.5, 9.0)	4.52 dd(3.5, 9.5)
4	5.63 d(3.5)	5.59 d(3.0)	5.64 d(3.5)	5.75 d(3.5)
5	4.35^a^	4.30^a^	4.26 q(6.0)	4.35 q(6.0)
6	1.47 d(6.0)	1.45 d(6.0)	1.46 d(6.0)	1.41 d(6.0)
Qui'-1	5.12 d(7.5)	5.14 d(8.5)	5.17 d(7.5)	5.14 d(8.0)
2	4.04^a^	4.05^a^	4.05^a^	4.11 dd(8.0, 9.0)
3	4.17^a^	4.25 dd(9.0, 9.0)	4.40 dd(9.5, 9.5)	4.22^a^
4	3.73^a^	5.34 dd(9.0, 9.0)	5.36 dd(9.5, 9.5)	5.34 dd(9.0, 9.0)
5	3.95 dq(9.5, 6.0)	4.06^a^	4.10 dq(9.5, 5.5)	4.00^a^
6	1.83 d(6.0)	1.67 d(6.0)	1.69 d(5.5)	1.64 d(6.0)
Rha'-1				5.95 br s
2				4.66^a^
3				4.40^a^
4				4.22^a^
5				4.44^a^
6				1.61 d(6.0)
Qui'''-1			5.14 d(8.0)	
2			3.90 dd(8.0, 9.5)	
3			4.07 dd(9.5, 9.5)	
4			3.61 dd(9.5, 9.5)	
5			3.71 dq(9.5, 6.0)	
6			1.56 d(6.0)	
Ipa-2	2.73 dd(7.5, 15.5)	2.73 dd(8.0, 16.0)	2.73 dd(8.5, 15.5)	2.73 dd(7.5, 15.5)
2	2.71 dd(5.0, 15.5)	2.70 dd(4.5, 16.0)	2.70 dd(5.0, 15.5)	2.70 dd(5.5, 15.5)
3	4.42^a^	4.41^a^	4.40^a^	4.41^a^
11	3.85^a^	3.86 m	3.85 m	3.79^a^
14	0.93 t(7.0)	0.96 t(7.0)	0.97 t(7.0)	0.95 t(7.0)
OCH_3_	3.64 s	3.63 s	3.63 s	3.63 s
Hda-2	2.61 ddd(8.0, 9.0, 15.5)	2.58^a^	2.56 m	2.57^a^
2	2.51 ddd(7.0, 9.0, 15.5)	2.51^a^		2.50^a^
7	3.88^a^	3.90 m	3.91^a^	3.90^a^
10	0.97 t(7.0)	0.97 t(7.0)	0.97 t(7.0)	0.96 t(7.0)
Hda'-2				2.33^a^
2				2.32^a^
7				3.90^a^
10				0.96 t(7.0)
Qui"-1	4.80 d(7.5)	4.81 d(7.5)	4.81 d(8.0)	4.81 d(7.5)
2	3.98^a^	3.98 dd(7.5, 9.0)	3.97 dd(8.0, 9.0)	3.98^a^
3	4.17^a^	4.16 dd(9.0, 9.0)	4.16 dd(9.0, 9.0)	4.16^a^
4	3.73^a^	3.73 dd(9.0, 9.0)	3.74 dd(9.0, 9.0)	3.73 dd(9.0, 9.0)
5	3.79 dq(9.5, 6.0)	3.79 dq(9.0, 6.0)	3.81^a^	3.80^a^
6	1.63 d(6.0)	1.64 d(6.0)	1.65 d(6.0)	1.65 d(6.0)
Qui''''-1				4.84 d(7.5)
2				3.98^a^
3				4.16^a^
4				3.72 dd(9.0, 9.0)
5				3.80^a^
6				1.66 d(6.0)
Nla-2	2.81 dq(7.5, 7.5)	2.82 dq(7.5, 7.5)	2.82 dq(7.5, 7.5)	
3	4.36^a^	4.36^a^	4.37^a^	
4	1.45 d(7.5)	1.49 d(7.0)	1.49 d(7.0)	
5	1.35 d(7.5)	1.37 d(7.5)	1.46 d(7.5)	
Mba-2		2.55 dq(7.0, 7.0, 7.0)		2.58 ddq(7.0, 7.0, 7.0)
3		1.85 m		1.84 m
3		1.55^a^		1.53^a^
4		1.01 dd(7.5, 7.5)		1.00 dd(7.5, 7.5)
5		1.25 d(7.0)		1.24 d(7.0)
Tig-3			7.17 qq(1.0, 7.0)	
4			1.74 d(7.0)	
5			2.01 q(1.0)	

*δ* in ppm from TMS [coupling constants (*J*) in Hz are given in parentheses]

*Qui* quinovopyranosyl, *Glc* glucopyranosyl, *Rha* rhamnopyranosyl, *Fuc* fucopyranosyl, *Ipa* methyl ipurolate moiety, *Hda* 7-hydroxydecanoyl, *Nl*a niloyl, *Mba* 2-methylbutyl, *Tig* tigloyl

^a^Signals were overlapped with other signals

**Table 2:** ^13^C-NMR spectroscopic data for **1**–**4** (in pyridine-*d*_5_, 150 MHz)

Position	**1**	**2**	**3**	**4**	**P**osition	**1**	**2**	**3**	**4**
Qui-1	102.7	102.7	102.7	102.5	Ipa-8	30.1^a^	30.1^a^	30.1^a^	30.1^a^
2	80.3	80.3	80.3	80.8	9	25.1^b^	25.1^b^	25.0^b^	25.1^b^
3	78.7	78.6	78.7	78.8	10	34.7^c^	34.7	34.7	34.7^c^
4	77.0	77.0	77.0	77.0	11	80.8	80.8	80.8	80.9
5	72.7	72.7	72.5	72.5	12	37.7	37.7	37.7	37.7^d^
6	18.2	18.2	18.5	18.6	13	18.8^d^	18.8^c^	18.8^d^	18.8^e^
Glc-1	101.7	101.6	101.5	101.8	14	14.4	14.5	14.4	14.5
2	76.0	75.9	75.9	75.6	OCH_3_	51.3	51.3	51.3	51.3
3	79.1	79.2	79.1	79.1	Hda-1	173.7	173.4	173.4	173.4
4	72.5	71.6	71.7	71.6	2	34.7^c^	34.7	34.7	34.8^c^
5	77.6	77.6	77.9	77.9	3	25.2^b^	25.3^b^	25.3^b^	25.3^b^
6	63.1	63.1	63.1	63.1	4	29.9^a^	30.0^a^	30.0^a^	30.0^a^
Rha-1	96.8	96.9	96.6	96.9	5	25.4^b^	25.5^b^	25.4^b^	25.4^b^
2	73.8	74.0	74.1	74.0	6	34.6^c^	34.7	34.7	34.8^c^
3	74.2	73.6	73.5	74.0	7	78.8	78.6	78.6	78.6
4	79.7	80.0	80.0	79.6	8	37.4	37.4	37.4	37.4
5	67.5	67.4	67.4	67.5	9	18.9^d^	18.9^c^	18.9^d^	18.9^e^
6	19.0	19.0	19.0	19.0	10	14.5	14.5	14.4	14.4
Glc'-1	101.2	101.2	101.3	101.3	Qui"-1	103.5	103.4	103.5	103.4
2	85.4	85.9	86.4	85.7	2	75.7	75.6	75.6	75.6
3	77.0	77.0	77.2	76.9	3	78.2	78.3	78.3	78.2
4	72.6	72.5	72.8	72.4	4	77.0	76.8	77.0	77.0
5	78.1	78.1	78.0	78.3	5	72.7	72.8	72.8	72.8
6	62.9	62.9	62.8	62.8	6	18.7	18.8	18.7	18.7
Qui'-1	106.2	106.6	106.5	106.7	Hda'-1				173.4
2	77.0	77.4	77.6	77.6	2				34.9^c^
3	78.2	75.6	83.4	75.6	3				25.6^b^
4	76.8	76.7	74.8	76.6	4				29.7^a^
5	73.8	70.8	71.7	70.1	5				24.8^b^
6	18.9	18.6	18.2	18.7	6				34.5^c^
Fuc-1	102.1	101.9	101.9	102.2	7				78.6
2	74.2	74.1	74.1	73.8	8				37.8^d^
3	72.7	72.5	72.7	77.2	9				18.9
4	74.1	74.0	73.9	73.5	10				14.4
5	69.2	69.1	69.1	69.3	Qui''''-1				103.5
6	16.7	16.7	16.7	16.7	2				75.6
Rha'-1				103.4	3				78.3
2				72.4	4				77.0
3				72.5	5				72.8
4				74.1	6				18.7
5				69.3	Nla-1	174.8	174.7	174.7	
6				18.7	2	50.6	50.7	50.7	
Qui'''-1			105.9		3	69.4	70.0	70.0	
2			76.3		4	20.8	20.8	20.9	
3			78.6		5	14.5	14.5	14.4	
4			76.6		Mba-1		176.0		176.0
5			73.2		2		41.8		41.8
6			18.5		3		27.3		27.1
Ipa-1	172.9	172.9	172.9	172.9	4		12.0		12.0
2	43.4	43.4	43.4	43.4	5		17.1		17.1
3	68.2	68.2	68.2	68.2	Tig-1			167.3	
4	38.2	38.2	38.2	38.2	2			129.1	
5	26.3	26.3	26.3	26.3	3			137.5	
6	30.6^a^	30.6^a^	30.6^a^	30.6^a^	4			14.2	
7	30.3^a^	30.3^a^	30.3^a^	30.3^a^	5			12.6	

*δ* in ppm from TMS

*Qui* quinovopyranosyl, *Glc* glucopyranosyl, *Rha* rhamnopyranosyl, *Fuc* fucopyranosyl, *Ipa* methyl ipurolate moiety, *Hda* 7-hydroxydecanoyl, *Nla* niloyl, *Mba* 2-methylbutyl, *Tig* tigloyl

^a,b,c,d,e^The assignments may be interchanged between the respective symbols in each compound

Using ESI-TOF-MS, the molecular formula of **2** (lacunosin II) was determined to be C_77_H_134_O_39_ by HR-positive-ion and HR-negative-ion modes. The ^1^H- and ^13^C-NMR spectra of **2** were similar to those of **1**, except for additional signals due to the presence of 1 mol of the 2-methylbutyryl residue (Mba) (Tables 1 and 2). The absolute configuration of the 2-methylbutyric acid component in the crude resin glycoside fraction derived from *I. lacunosa* seeds was determined to be *S*, following the same procedure as those used for nilic acid [10]. The NMR signals were assigned using the same 2D-NMR spectroscopy used for **1**. A comparison of the ^1^H-NMR signals corresponding to the sugar moieties of **2** and **1** revealed a downfield shift (1.61 ppm) in the signal corresponding to H-4 of Qui' in **2**. The other signals, including H-2 of Rha and H-4 of Fuc, which are indicative of acylation shifts, showed chemical shifts almost identical to those of **1**. In addition, **2** exhibits significant correlations in the HMBC spectrum between the signals of H-1 of Qui and C-11 of Ipa; H-1 of Glc and C-2 of Qui; H-3 of Rha and C-1 of Glc'; H-4 of Rha and C-1 of Fuc; H-1 of Qui' and C-2 of Glc'; H-1 of Qui'' and C-7 of Hda; the methoxy protons and C-1 of Ipa; H-2 of Rha and C-1 of Hda; H-4 of Fuc and C-1 of Nla; and H-4 of Qui' and C-1 of Mba (Fig. 1). No HMBC correlations were observed to confirm the sugar linkage between Glc and Rha. However, significant correlations were identified in the NOESY spectrum of **2**, including cross-peaks between the signals of H-1 of Qui and H-11 of Ipa; H-1 of Glc and H-2 of Qui; H-1 of Rha and H-2 of Glc; H-1 of Glc' and H-3 of Rha; H-1 of Fuc and H-4 of Rha; and H-1 of Qui' and H-2 of Glc' (Fig. 1). Moreover, although no HMBC correlation was detected between H-2 of Rha and C-1 of Hda, the observed acylation-induced shift in the signals suggested that Hda was esterified at C-2 of Rha. Consequently, **2** was concluded to be a homolog of **1** in which Mba was bonded to C-4 of Qui' (Fig. 2).

HR-positive-ion and HR-negative-ion ESI-TOF–MS data for **3** (lacunosin III) revealed that the molecular formula of **3** is C_83_H_142_O_43_. The ^1^H- and ^13^C-NMR signals of **3**, assigned using the same 2D-NMR spectroscopic techniques as **1**, were similar to those of **2**, especially those of Agl, which were nearly identical. However, additional signals corresponding to 1 mol each of the tigloyl residue and *β*-quinovopyranosyl residue with ^4^C_1_ conformation were observed, along with the disappearance of signals corresponding to Mba (Tables 1 and 2). Tiglic acid was identified as an organic acid component in the crude resin glycoside fraction of *I. lacunosa* seeds; however, its geometrical isomer, angelic acid, was not detected. By comparing the ^13^C-NMR signals of the sugar moieties between **3** and **2**, downfield shifts (∆*δ* = *δ***3**–*δ***2**) were observed at the signals attributed to C-3 (∆*δ* = 7.8) and C-5 (∆*δ* = 0.9) of Qui', and an upfield shift was observed at that attributed to C-4 (∆*δ* = − 1.9) of Qui' [14, 15]. The other signals are nearly identical. In addition, acylation shifts were observed for the same signals as those observed for **2**. In the HMBC spectrum of **3**, important cross-peaks were detected between the signals of H-1 of Qui and C-11 of Ipa; H-1 of Glc and C-2 of Qui; H-4 of Rha and C-1 of Fuc; H-1 of Qui' and C-2 of Glc'; H-1 of the fourth quinovosyl residue (Qui''') and C-3 of Qui'; H-4 of Fuc and C-1 of Nla; H-4 of Qui' and C-1 of the tigloyl residue (Tig); H-1 of Qui'' and C-7 of Hda; and methoxy protons and C-1 of Ipa (Fig. 1). No valid HMBC correlation was detected between H-2 of Rha and C-1 of Hda, nor was any correlation detected to confirm the sugar linkages at the anomeric positions of Rha and Glc'. However, the ester linkage of Hda was determined based on the observed acylation shifts, leading to the conclusion that the carboxyl group of Hda is bound to C-2 of Rha. Furthermore, the sugar linkages at the anomeric positions of Rha and Glc' were elucidated by following the NOESY correlations, which indicated that Rha is attached to C-2 of Glc and Glc' is attached to C-3 of Rha. These correlations included the following cross-peaks: between H-1 of Qui and H-11 of Ipa; H-1 of Glc and H-2 of Qui; H-1 of Rha and H-2 of Glc; H-1 of Glc' and H-3 of Rha; H-1 of Fuc and H-4 of Rha; H-1 of Qui' and H-2 of Glc'; and H-1 of Qui''' and H-3 of Qui' (Fig. 1). Thus, **3** was identified as methyl 3*S*,11*S*-ipurolate 11-*O*-*β*-d-quinovopyranosyl-(1 → 3)-*O*-(4-*O*-tigloyl)-*β*-d-quinovopyranosyl-(1 → 2)-*O*-β-d-glucopyranosyl-(1 → 3)-[-*O*-(4-*O*-2*R*,3*R*-niloyl)-*β*-d-fucopyranosyl-(1 → 4)]-*O*-(2-*O*-7*S*-hydroxydecanoyl 7-*O*-*β*-d-quinovopyranoside)-*α*-l-rhamnopyranosyl-(1 → 2)-*O*-*β*-d-glucopyranosyl-(1 → 2)-*β*-d-quinovopyranosyide (Fig. 2). Compound **3** contained a new glycosidic acid, an isomer of lacunosinic acid H [10], in which the terminal rhamnosyl residue was substituted with Qui'''.

The molecular formula of **4** (lacunosin IV) was determined to be C_94_H_164_O_47_ using the HR-positive-ion and HR-negative-ion modes in ESI-TOF–MS. The ^1^H- and ^13^C-NMR spectral data of **4**, assigned using 2D-NMR spectroscopy, were similar to those of **2**. In particular, the data for Ipa were nearly identical. However, additional signals attributed to 1 mol each of *α*-rhamnopyranosyl and quamoclinic acid B residues are observed, along with the absence of signals corresponding to 1 mol of niloyl residue in **4** (Tables 1 and 2). A comparison of the ^13^C-NMR signals of **4** and **2** revealed a downfield shift in the signal attributed to C-3 (4.7 ppm) of Fuc in **4**. In contrast, the signals corresponding to the other sugar moieties in **2** were nearly identical to those observed in **4**. The HMBC spectrum of **4** reveals significant correlations, including H-1 of Glc with C-2 of Qui; H-2 of Glc with C-1 of Rha; H-3 of Rha with C-1 of Glc'; H-4 of Rha with C-1 of Fuc; H-1 of Qui' with C-2 of Glc'; H-3 of Fuc with C-1 of Rha', C-1 of Qui'', or C-1 of the fifth quinovosyl residue (Qui''''); H-1 of Qui'' with C-7 of Hda; and H-1 of Qui'''' with C-7 of the second 7-hydroxydecanoyl residue (Hda'; Agl of the second quamoclinic acid B residue); and methoxy protons with C-1 of Ipa (Fig. 1). Although no HMBC correlations suitable for determining the sugar linkages at the anomeric positions of Qui and Rha' were observed, the NOESY spectrum of **4** revealed notable cross-peaks, including those between the signals of H-1 of Qui and H-11 of Ipa; H-1 of Glc and H-2 of Qui; H-1 of Rha and H-2 of Glc; and H-1 of Fuc and H-4 of Rha. In addition, a prominent cross-peak was observed between H-1 of the second rhamnosyl residue (Rha') and H-3 of Fuc (Fig. 1). These data indicate that **4** contains 1 mol of 2-methylbutyric acid, 2 mol of quamoclinic acid B, and 1 mol of new glycosidic acid methyl ester, identified as methyl 3*S*,11*S*-ipurolate 11-*O*-*β*-d-quinovopyranosyl-(1 → 2)-*O*-*β*-d-glucopyranosyl-(1 → 3)-[*O*-*α*-l-rhamnopyranosyl-(1 → 3)-*O*-*β*-d-fucopyranosyl-(1 → 4)]-*O*-*α*-l-rhamnopyranosyl-(1 → 2)-*O*-*β*-d-glucopyranosyl-(1 → 2)-*β*-d-quinovopyranoside. This new glycosidic acid methyl ester was found to be a homolog of the lacunosinic acid D methyl ester (**8**) [10], in which the aglycone methyl 3*S*,11*S*-dihydroxyhexadecanoate was substituted with methyl 3*S*,11*S*-ipurolate. Comparing the ^1^H-NMR spectral data corresponding to sugar moieties of **4** and **8** [10], downfield shifts are observed for the signals corresponding to H-2 (1.06 ppm) of Rha, H-4 (1.54 ppm) of Fuc, and H-4 (1.67 ppm) of Qui' in **4**. In addition, key HMBC correlations were observed between the signals of H-4 of Fuc and C-1 of Hda'; and H-4 of Qui' and C-1 of Mba (Fig. 1). These data indicate that Hda, Hda', and Mba are attached to C-2 of Rha, C-4 of Fuc, and C-4 of Qui', respectively. Consequently, **4** was concluded to be methyl 3*S*,11*S*-ipurolate 11-*O*-(4-*O*-2*S-*methylbutyryl)-*β*-d-quinovopyranosyl-(1 → 2)-*O*-*β*-d-glucopyranosyl-(1 → 3)-[*O*-*α*-l-rhamnopyranosyl-(1 → 3)-*O*-(4-*O*-7*S*-hydroxydecanoyl 7-*O*-*β*-d-quinovopyranoside)-*β*-d-fucopyranosyl-(1 → 4)]-*O*-(2-*O*-7*S*-hydroxydecanoyl 7-*O*-*β*-d-quinovopyranoside)-*α*-l-rhamnopyranosyl-(1 → 2)-*O*-*β*-d-glucopyranosyl-(1 → 2)-*β*-d-quinovopyranoside (Fig. 2).

The HR-negative-ion ESI-TOF-MS and NMR data revealed that the molecular formula of **5** (lacunosinic acid I) was C_34_H_62_O_18._ The ^1^H-NMR spectrum of **5** showed signals corresponding to three anomeric protons, two secondary methyl groups, two non-equivalent methylene protons adjacent to the carbonyl group, and one primary methyl group. The ^13^C-NMR spectrum shows signals attributed to one carboxyl carbon and three anomeric carbons, along with 30 aliphatic carbons. NMR assignments were performed using the same 2D-NMR spectroscopies as those employed for **1** (Table 3). These data suggest that **5** consists of 1 mol of *α*-l-rhamnopyranose with ^1^C_4_ conformation and 2 mol of *β*-d-glucopyranose with ^4^C_1_ conformation as monosaccharides, and dihydroxyhexadecanoic acid as the aglycone. The planar structure of the aglycone was determined from the correlations observed in the 2D-NMRspectra of **5**. In the ^1^H-^1^H COSY spectrum of **5**, significant correlations were observed sequentially from the H_2_-2 signals of Agl to H_2_-3 of Agl/H-4 (*δ* 4.94) of Agl (Fig. 1). In addition, HMBC correlations were observed between the signals of H_2_-2 of Agl and C-4 (*δ* 81.0) of Agl, indicating the presence of a hydroxyl group at C-4 (Fig. 1). The chemical shift of the signal attributed to the *β*-carbon of Agl in **5** was *δ* 32.4, suggesting that the remaining hydroxyl group was located at C-11. Thus, the aglycone of **5** is identified as 4,11-dihydroxyhexadecanoic acid, which is a novel compound. Comparison of the ^13^C-NMR data of **5** with those of the corresponding methyl pyranosides [21] revealed glycosylation shifts [14, 15] at the C-2 (+ 4.9 ppm) of the third glucosyl residue (Glc'') and C-2 (+ 4.5 ppm) of Glc. In the HMBC spectrum of **5**, key cross-peaks were detected between the signals of H-1 of Glc'' and C-11 of Agl; H-1 of Glc and C-2 of Glc''; and H-1 of Rha and C-2 of Glc (Fig. 1). In addition, the NOESY spectrum provided important correlations between the signals of H-1 of Glc and H-2 of Glc''; and H-1 of Rha and H-2 of Glc (Fig. 1). The absolute configurations of the monosaccharide components of the crude resin glycoside fraction obtained from *I. lacunosa* seeds have already been determined [10]. However, that of the aglycone in **5** could not be determined because of the yield of **5**. Therefore, **5** was identified as 4,11-dihydroxyhexadecanoic acid 11-*O*-*α*-l-rhamnopyranosyl-(1 → 2)-*O*-*β*-d-glucopyranosyl-(1 → 2)-*O*-*β*-d-glucopyranoside.

**Table 3:** ^1^H- and ^13^C-NMR spectroscopic data for **5** (in pyridine-*d*_5_)

Glc"-1	5.01 d(7.5)	102.8	Agl-1		177.4
2	4.38 dd(7.5, 9.0)	79.7	2	2.47 dd(7.0, 9.5)	29.1
3	4.54 dd(9.0, 9.0)	79.5	3	2.14 dddd(7.0, 7.0, 7.0, 13.0)	28.2
4	4.14 dd(9.0, 9.0)	72.0	3	1.67^a^	
5	3.92 ddd(2.5, 5.5, 9.0)	77.9	4	4.94^a^	81.0
6	4.49 dd(2.5, 11.5)	62.9	5	1.69^a^	34.6
6	4.35 dd(5.5, 11.5)		5	1.52^a^	
Glc'-1	5.88 d(7.5)	102.0	6	1.44^a^	25.7
2	4.26 dd(7.5, 9.0)	79.3	6	1.36^a^	
3	4.23 dd(9.0, 9.0)	78.8	7	1.44^a^	29.9
4	4.11 dd(9.0, 9.0)	72.7	7	1.27^a^	
5	3.85 ddd(3.5, 5.5, 9.0)	77.5	8	1.44^a^	30.4
6	4.43^a^	63.2	8	1.33^a^	
6	4.29 dd(5.5, 11.5)		9	1.77^a^	25.2
Rha-1	6.33 d(2.0)	102.2	10	1.77^a^	35.5
2	4.75 dd(2.0, 3.5)	72.4	11	3.99^a^	80.9
3	4.67 dd(3.5, 9.5)	72.6	12	1.88^a^	35.7
4	4.32 dd(9.5, 9.5)	74.4	12	1.68^a^	
5	5.02 dq(9.5, 6.0)	69.6	13	1.50^a^	25.4
6	1.82 d(6.0)	19.1	14	1.25^a^	32.4
			14	1.23^a^	
			15	1.26^a^	23.0
			16	0.82 t(7.0)	14.3

*δ* in ppm from TMS [coupling constants (*J*) in Hz are given in parentheses)]

*Glc* glucopyranosyl, *Rha* rhamnopyranosyl, *Agl* Aglycone moiety

The assignments of Agl-7 and Agl-8 may be interchanged

^1^H-NMR spectroscopic data were collected at 600 MHz

^13^C-NMR spectroscopic data were collected at 150 MHz

^a^Signals were overlapped with other signals

## Conclusion

In the present study, four new acylated glycosidic acid methyl esters (**1**–**4**) and one glycosidic acid (**5**) with a new aglycone, 4,11-dihydroxyhexadecanoic acid, were isolated from *I. lacunosa* seeds. Compounds **1** and **2** were heptaglycosides, **3** was an octaglycoside, and **4** was a nonaglycoside with methyl 3*S*,11*S*-dihydroxytetradecanoate as a common aglycone. In addition, **1**–**3** contained two glycosidic acids, and **4** had three glycosidic acids. Compound **5** is the first glycosidic acid with a hydroxyl group at C-4 of Agl. However, **1–4** may be artifacts produced during the extraction and/or isolation of the resin glycosides in the free carboxyl form (convolvulin) [1].

## Experimental

### General procedure

Optical rotations were measured using a P-2300 polarimeter (JASCO, Tokyo, Japan). ESI-TOF-MS was performed on a maXis II ETD instrument (Bruker, Billerica, MA, USA). NMR spectra were recorded using a JEOL ECZ-600R/S1 spectrometer (JEOL, Tokyo, Japan), and chemical shifts were reported on a *δ* (ppm) scale with tetramethylsilane (TMS) as the internal standard. Diaion HP20 (Mitsubishi Chemical Industries Co., Ltd., Tokyo, Japan), Sephadex LH-20 (Pharmacia Fine Chemicals, Uppsala, Sweden), silica gel 60 (Merck, Art. 1.09385; Merck, Darmstadt, Germany), Chromatorex C18 SMB (Fuji Silysia Chemical, Ltd., Aichi, Japan), and Chromatorex ODS column (Fuji Silysia Chemical, Ltd.) were used for column chromatography. HPLC separation was performed using a Shimadzu LC-20AT micropump (Shimadzu, Kyoto, Japan) with a Shimadzu RID-20A RI detector (Shimadzu). For the HPLC column, COSMOSIL 5C18-AR-II (Nacalai Tesque, Inc., Kyoto, Japan; 250 mm × 20 mm i.d.) was used.

### Plant material

The seeds of *Ipomoea lacunosa* were collected from the medical plant garden (33°32′59ʺ402N 130°21′47ʺ415E) of Fukuoka University, Fukuoka Prefecture, Japan, in September 2016, and identified by one of the authors (Prof. Okawa M.). A voucher specimen of the seeds (ILSFU2016) was deposited in the Laboratory of Natural Products Chemistry, School of Agriculture, Tokai University.

### Isolation of 1–5

The powdered seeds of *I. lacunosa* (993.34 g) were extracted with MeOH (1200, 900, 1000 ml) at room temperature for 106 days, and the solvent was removed under reduced pressure to afford a MeOH extract (73.74 g). The MeOH extract was partitioned between hexane (600 mL) and 95% MeOH (800 mL) to yield a hexane-soluble fraction (fr.) (8.55 g) and a 95% MeOH-soluble fr. (64.22 g). The 95% MeOH-soluble fr. was chromatographed over Diaion HP20 column eluted with 40% MeOH, 100% MeOH, and acetone to furnish 100% MeOH-eluated fr. (28.27 g) and acetone-eluated fr. (441 mg). The MeOH-eluated fr. was subjected to Sephadex LH-20 column chromatography eluted with MeOH to yield fr. 1 (6.706 g), fr. 2 (5.927 g), fr. 3 (5.324 g), and fr. 4 (7.744 g). Column chromatography of fr. 2 on silica gel eluted with a gradient of mixtures of CHCl_3_–MeOH–H_2_O (14:2:0.1, 10:2:0.1, 8:2:0.2, 7:3:0.5, 6:4:1) afforded fractions (frs.) 2-1–2-13. Fraction 2-5 (560 mg) was chromatographed on a Chromatorex C18 SMB column eluted with a gradient of mixtures of H_2_O–MeOH (80% MeOH, 85% MeOH, 90% MeOH, 95% MeOH, 100% MeOH) to yield frs 2-5-1–2-5-10. HPLC of fr. 25-7 (160 mg) eluted with 85% MeOH afforded **2** (7 mg). Fraction 2-7 (742 mg) was chromatographed on a Chromatorex ODS column eluted with a gradient of mixtures of H_2_O–MeOH (70% MeOH, 75% MeOH, 80% MeOH, 85% MeOH, 90% MeOH, 95% MeOH, 100% MeOH) furnished frs. 2-7-1–2-7-13. Fractions 2-7-5 (10 mg) and 2-7-9 (114 mg) were each subjected to HPLC eluted with 75% MeOH for fr. 2-7-5 and 85% MeOH for fr. 2-7-9 to give **5** (4 mg) from fr. 2-7-5 and **1** (6 mg), **3** (5 mg), and **4** (13 mg) from fr. 2-7-9.

Lacunosin I (**1**). Amorphous powder. [*α*]^24^_D_–38.4° (*c* = 0.4, MeOH). HR-positive-ion ESI-TOF–MS *m/z*: 1621.7816 [M + Na]^+^ (Calcd for C_72_H_126_NaO_38_^+^, 1621.7819). HR-negative-ion ESI-TOF–MS *m/z*: 1597.7852 [M–H]^–^ (Calcd for C_72_H_125_O_38_^–^, 1597.7854). HR-negative-ion ESI-TOF–MS/MS *m/z*: 1281.5956 (Calcd for C_56_H_97_O_32_^–^, 1281.5963). ^1^H-NMR spectral data: see Table 1. ^13^C-NMR spectral data: see Table 2. ^1^*J*_C-1–H-1_ (Hz): Qui (156.2), Glc (168.3), Rha (178.2), Glc' (169.5), Fuc (170.7), Qui' (157.8), Qui'' (159.1).

Lacunosin II (**2**). Amorphous powder. [*α*]^24^_D_–36.6° (*c* = 0.6, MeOH). HR-positive-ion ESI-TOF–MS *m/z*: 1705.8358 [M + Na]^+^ (Calcd for C_77_H_134_NaO_39_^+^, 1705.8394). HR-negative-ion ESI-TOF–MS *m/z*: 1681.8396 [M–H]^–^ (Calcd for C_77_H_133_O_39_^–^, 1681.8429). ^1^H-NMR spectral data: see Table 1. ^13^C-NMR spectral data: see Table 2.

Lacunosin III (**3**). Amorphous powder. [*α*]^24^_D_–38.4° (*c* = 0.4, MeOH). HR-positive-ion ESI-TOF–MS (+ HCOONH_4_) *m/z*: 1844.9204 [M + NH_4_]^+^ (Calcd for C_83_H_146_NO_43_^+^, 1844.9263). HR-negative-ion ESI-TOF-MS *m/z*: 1825.8811 [M–H]^–^ (Calcd for C_83_H_141_O_43_^–^, 1825.8852). ^1^H-NMR spectral data: see Table 1. ^13^C-NMR spectral data: see Table 2.

Lacunosin IV (**4**). Amorphous powder. [*α*]^24^_D_–54.4° (*c* = 0.4, MeOH). HR-positive-ion ESI-TOF–MS (+ HCOONH_4_) *m/z*: 2064.0840 [M + NH_4_]^+^ (Calcd for C_94_H_168_NO_47_^+^, 2064.0815). HR-negative-ion ESI-TOF–MS *m/z*: 2045.0353 [M–H]^–^ (Calcd for C_94_H_163_O_47_^–^, 2045.0403). ^1^H-NMR spectral data: see Table 1. ^13^C-NMR spectral data: see Table 2.

Lacunosinic acid I (**5**). Amorphous powder. [*α*]^25^_D_–47.2° (*c* = 0.3, MeOH). HR-negative-ion ESI-TOF–MS *m/z*: 739.3750 [M–H_2_O–H]^–^ (Calcd for C_34_H_59_O_17_^–^, 739.3758). HR-negative-ion ESI-TOF–MS (+ HCOONH_4_) *m/z*: 785.3809 [M–H_2_O + HCOO]^–^ (Calcd for C_35_H_61_O_19_^–^, 785.3813). ^1^H-NMR spectral data: see Table 3. ^13^C-NMR spectral data: see Table 3.

## Supplementary Information

Below is the link to the electronic supplementary material.

Supplementary file1 (PDF 25547 KB)

## References

[1] Ono M (2017) Resin glycosides from Convolvulaceae plants. J Nat Med 71:591–604 28748432 10.1007/s11418-017-1114-5 PMC6763574

[2] Pereda-Miranda R, Rosas-Ramírez D, Castańeda-Gómez J (2010) Resin glycosides from morning glory family. In: Kinghorn A D, Falk H, Kobayashi J (eds) Progress in the chemistry of organic natural products. Springer-Verlag 92:77–153 10.1007/978-3-211-99661-4_2 20198465

[3] Ono M, Tenmaya D, Tarumi M, Satou S, Kotone T, Nishikawa H, Yasuda S, Hi M, Zhou JR, Yokomizo K, Yoshimitsu H, Tsuchihashi R, Okawa M, Kinjo J (2024) Four new resin glycosides from *Ipomoea muricata* seeds: Muricatins XIV–XVII. J Nat Med 78:525–536 38457082 10.1007/s11418-024-01787-1

[4] Ono M, Taketomi S, Kakiki Y, Nishikawa H, Yauda S, Tsuchihashi R, Okawa M, Kinjo J, Miyashita H, Yoshimitsu H, Nohara T (2024) Two new resin glycosides, muricatins XII and XIII, from the seeds of *Ipomoea muricata*. Nat Prod Res 38:423–432 36148550 10.1080/14786419.2022.2125970

[5] Yoshikawa K, Yagi C, Hama H, Tanaka M, Arihara S, Hashimoto T (2010) Ipomotaosides A-D, resin glycosides from the aerial parts of *Ipomoea batatas* and their inhibitory activity against COX-1 and COX-2. J Nat Prod 73:1763–1766 20961090 10.1021/np100283t

[6] Figueroa-González G, Jacobo-Herrera N, Zentella-Dehesa A, Pereda-Miranda R (2012) Reversal of multidrug resistance by morning glory resin glycosides in human breast cancer cells. J Nat Prod 75:93–97 22148475 10.1021/np200864m

[7] Ono M, Takigawa A, Kanemaru Y, Kawakami G, Kabata K, Okawa M, Kinjo J, Yokomizo K, Yoshimitsu H, Nohara T (2014) Calysolins V-IX, resin glycosides from *Calystegia soldanella* and their antiviral activity toward herpes. Chem Pharm Bull 62:97–105 10.1248/cpb.c13-00610 24390499

[8] Ribeiro DN, Nandula VK, Dayan FE, Rimando AM, Duke SO, Reddy KN, Shaw DR (2015) Possible glyphosate tolerance mechanism in pitted morningglory (*Ipomoea lacunosa* L.). J Agric Food Chem 63:1689–1697 25625294 10.1021/jf5055722

[9] Wagner H, Schwarting G, Varljen J, Bauer R, Hamdard ME, El-Faer MZ, Bea J (1983) Chemical constituents of the Convolvulaceae resins. IV. Glycosidic acids of *Ipomoea quamoclit*, *I. lacunosa*. I pandurata Convolvulusal-sirensis Planta Medica 49:154–157 17405039 10.1055/s-2007-969837

[10] Uemura K, Murakami R, Kimura E, Kai M, Misuda N, Yasuda S, Miyashita H, Yoshimitsu H, Tsuchihasi R, Okawa M, Kinjo J, Ono M (2024) Identification and characterization of organic and glycosidic acids in the crude resin glycoside fraction of *Ipomoea lacunosa* seeds. Carbohydr Res. 10.1016/j.carres.2024.109048 38310808 10.1016/j.carres.2024.109048

[11] Misuda N, Nishikawa H, Yasuda S, Miyashita H, Yoshimitsu H, Tsuchihashi R, Okawa M, Kinjo J, Ono M (2024) Eleven new glycosidic acid methyl esters from the crude resin glycoside fraction of *Ipomoea alba* seeds. J Nat Med 78:1057–1070 39158815 10.1007/s11418-024-01838-7

[12] Kasai R, Okihara M, Asakawa J, Mizutani K, Tanaka O (1977) ^13^C NMR study of *α*- and *β*-anomeric pairs of d-mannopyranosides and l-rhamnopyranosides. Tetrahedron 1977:175–178

[13] Uemura K, Kimura S, Saito Y, Koyama S, Nishikawa H, Yasuda S, Miyashita H, Yoshimitsu H, Tsuchihashi R, Okawa M, Kinjo J, Ono M (2022) Identification and characterization of organic and glycosidic acids in the crude resin glycoside fraction from the leaves and stems of *Calystegia japonica*. J Nat Med 77:284–297 36527581 10.1007/s11418-022-01669-4

[14] Kasai R, Suzuo M, Asakawa J, Tanaka O (1977) Carbon-13 chemical shifts of isoprenoid-*β*-d-glucopyranosides and -*β*-d-mannopyranosides. Stereochemical influences of aglycone alcohols. Tetrahedron Lett 2:175–178

[15] Tori K, Seo S, Yoshimura Y, Arita H, Tomita Y (1977) Glycosidation shifts in carbon-13 NMR spectroscopy: carbon-13 signal shifts from aglycone and glucose to glucoside. Tetrahedron Lett 2:179–182

[16] Ono M, Noda N, Kawasaki T, Miyahara K (1990) Resin Glycosides. VII. reinvestigation of the component organic and glycosidic acids of pharbitin, the crude ether-insoluble resin glycoside (“convolvulin”) of Pharbitidis Semen (Seeds of *Pharbitis nil*). Chem Pharm Bull 38:1892–1897

[17] Ono M, Kishida M, Ikegami Y, Takaki Y, Okawa M, Kinjo J, Yoshimitsu H, Nohara T, Miyahara K (2011) Components of convolvulin from *Quamoclit* x *multifida*. J Nat Med 65:95–102 20835850 10.1007/s11418-010-0464-z

[18] Ono M, Takagi-Taki Y, Honda-Yamada F, Noda N, Miyahara K (2010) Components of ether-insoluble resin glycoside (Convolvulin) from Seeds of *Quamoclit pennata*. Chem Pharm Bull 58:666–672 10.1248/cpb.58.666 20460794

[19] Ono M, Akiyama K, Yamamoto K, Mineno T, Okawa M, Kinjo J, Miyashita H, Yoshimitsu H, Nohara T (2014) Four new acylated glycosidic acid methyl Esters isolated from the convolvulin fraction of seeds of *Quamoclit pennata* after treatment with indium(III) chloride in methanol. Chem Pharm Bull 62:830–835 10.1248/cpb.c14-00277 25087636

[20] Akiyama K, Mineno T, Okawa M, Kinjo J, Miyashita H, Yoshimitsu H, Nohara T, Ono M (2013) Three acylated glycosidic acid methyl esters and two acylated methyl glycosides generated from the convolvulin fraction of seeds of *Quamoclit pennata* by treatment with indium(III) chloride in methanol. Chem Pharm Bull 61:952–961 10.1248/cpb.c13-00355 23995359

[21] Seo S, Tomita Y, Tori K, Yoshimura Y (1978) Determination of the absolute configuration of a secondary hydroxy group in a chiral secondary alcohol using glycosidation shifts in carbon-13 nuclear magnetic resonance spectroscopy. J Am Chem Soc 100:3331–3339

