# Supplementary material for: Four new acylated glycosidic acid methyl esters and a new glycosidic acid from Ipomoea lacunosa seeds
Source: J Nat Med. 2025 Feb 13;79(2):422–34. doi: 10.1007/s11418-025-01877-8 (PMC11880051; doi:10.1007/s11418-025-01877-8)
Supplement: Supplementary file 1 — Supplementary file1 (PDF 25547 KB) [file 11418_2025_1877_MOESM1_ESM.pdf]

Supplementary data

Four new acylated glycosidic acid methyl esters and a new glycosidic acid from *Ipomoea lacunosa* seeds

Masateru Ono · Renjyu Murakami · Shin Yasuda · Hiroyuki Miyashita · Hitoshi Yoshimitsu · Ryota Tsuchihashi · Masafumi Okawa · Junei Kinjo

List of Content

Figure S1: HR-positive-ion ESI-TOF-MS of **1**
Figure S2: HR-negative-ion ESI-TOF-MS of **1**
Figure S3: HR-negative-ion ESI-TOF-MS/MS of $[M–H]^-$ ion of **1**
Figure S4: ^{1}H-NMR (600 MHz, pyridine-d_5) spectrum of **1**
Figure S5: ^{13}C-NMR (150 MHz, pyridine-d_5) spectrum of **1**
Figure S6: DEPT (150 MHz, pyridine-d_5) spectrum of **1**
Figure S7: ^{1}H-^{1}H COSY (600 MHz, pyridine-d_5) spectrum of **1**
Figure S8: ^{1}H-^{1}H TOCSY (600 MHz, pyridine-d_5) spectrum of **1**
Figure S9: HMQC (600 MHz, pyridine-d_5) spectrum of **1**
Figure S10: HSQC (600 MHz, pyridine-d_5) spectrum of **1**
Figure S11: HMBC (600 MHz, pyridine-d_5) spectrum of **1**
Figure S12: NOESY (600 MHz, pyridine-d_5) spectrum of **1**
Figure S13. HR-positive-ion ESI-TOF-MS of **2**
Figure S14: HR-negative-ion ESI-TOF-MS of **2**
Figure S15: ^{1}H-NMR (600 MHz, pyridine-d_5) spectrum of **2**
Figure S16: ^{13}C-NMR (150 MHz, pyridine-d_5) spectrum of **2**
Figure S17: DEPT (150 MHz, pyridine-d_5) spectrum of **2**
Figure S18: ^{1}H-^{1}H COSY (600 MHz, pyridine-d_5) spectrum of **2**
Figure S19: ^{1}H-^{1}H TOCSY (600 MHz, pyridine-d_5) spectrum of **2**
Figure S20: HMQC (600 MHz, pyridine-d_5) spectrum of **2**

Figure S21: HMBC (600 MHz, pyridine-d_5) spectrum of **2**

Figure S22: NOESY (600 MHz, pyridine-d_5) spectrum of **1**

Figure S23: HR-positive-ion ESI-TOF-MS (+$HCOONH_4$) of **3**

Figure S24: HR-negative-ion ESI-TOF-MS of **3**

Figure S25: ^{1}H-NMR (600 MHz, pyridine-d_5) spectrum of **3**

Figure S26: ^{13}C-NMR (150 MHz, pyridine-d_5) spectrum of **3**

Figure S27: DEPT (150 MHz, pyridine-d_5) spectrum of **3**

Figure S28: ^{1}H-^{1}H COSY (600 MHz, pyridine-d_5) spectrum of **3**

Figure S29: ^{1}H-^{1}H TOCSY (600 MHz, pyridine-d_5) spectrum of **3**

Figure S30: HMQC (600 MHz, pyridine-d_5) spectrum of **3**

Figure S31: HMBC (600 MHz, pyridine-d_5) spectrum of **3**

Figure S32: NOESY (600 MHz, pyridine-d_5) spectrum of **3**

Figure S33: HR-positive-ion ESI-TOF-MS of **4**

Figure S34: HR-negative-ion ESI-TOF-MS of **4**

Figure S35: ^{1}H-NMR (600 MHz, pyridine-d_5) spectrum of **4**

Figure S36: ^{13}C-NMR (150 MHz, pyridine-d_5) spectrum of **4**

Figure S37: DEPT (150 MHz, pyridine-d_5) spectrum of **4**

Figure S38: ^{1}H-^{1}H COSY (600 MHz, pyridine-d_5) spectrum of **4**

Figure S39: ^{1}H-^{1}H TOCSY (600 MHz, pyridine-d_5) spectrum of **4**

Figure S40: HMQC (600 MHz, pyridine-d_5) spectrum of **4**

Figure S41: HMBC (600 MHz, pyridine-d_5) spectrum of **4**

Figure S42: NOESY (600 MHz, pyridine-d_5) spectrum of **4**

Figure S43: HR-negative-ion ESI-TOF-MS of **5**

Figure S44: HR-negative-ion ESI-TOF-MS (+$HCOONH_4$) of **5**

Figure S45: ^{1}H-NMR (600 MHz, pyridine-d_5) spectrum of **5**

Figure S46: ^{13}C-NMR (150 MHz, pyridine-d_5) spectrum of **5**

Figure S47: DEPT (150 MHz, pyridine-d_5) spectrum of **5**

Figure S48: ^{1}H-^{1}H COSY (600 MHz, pyridine-d_5) spectrum of **5**

Figure S49: ^{1}H-^{1}H TOCSY (600 MHz, pyridine-d_5) spectrum of **5**

Figure S50: HMQC (600 MHz, pyridine-d_5) spectrum of **5**

Figure S51: HMBC (600 MHz, pyridine-d_5) spectrum of **5**

Figure S52: NOESY (600 MHz, pyridine-d_5) spectrum of **5**

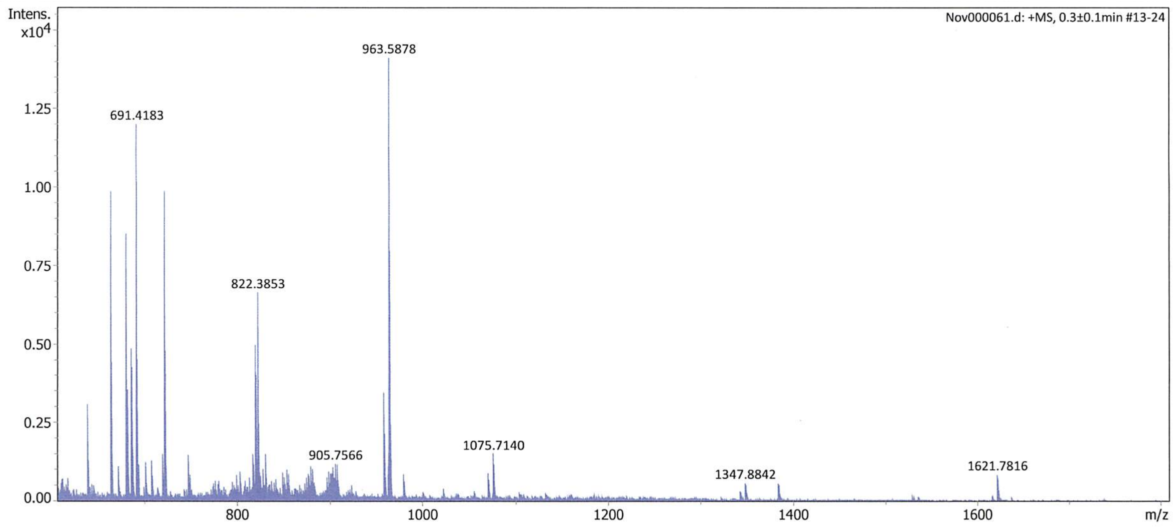

Figure S1: HR-positive-ion ESI-TOF-MS of **1**

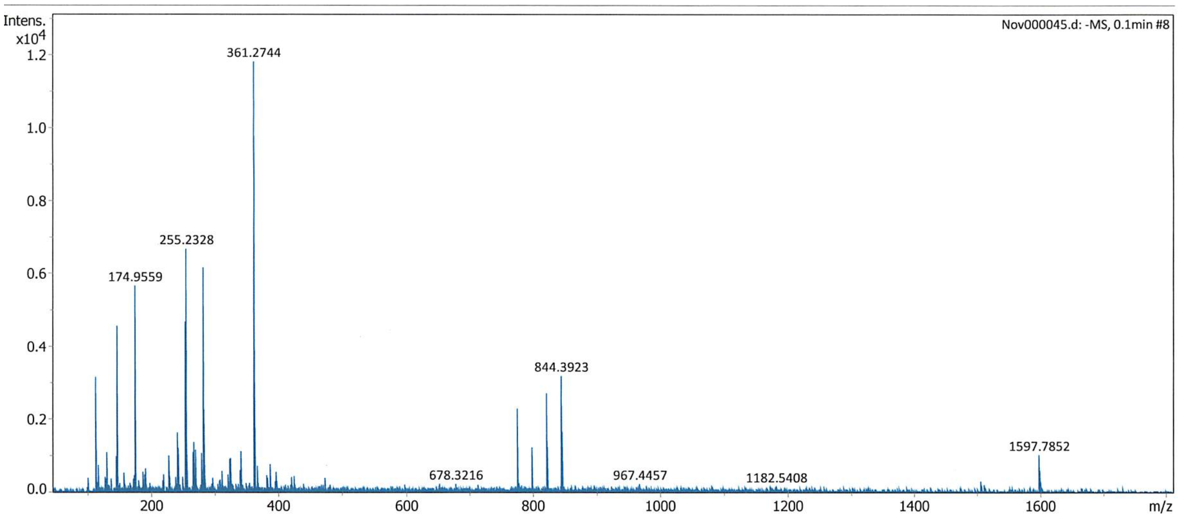

Figure S2: HR-negative-ion ESI-TOF-MS of **1**

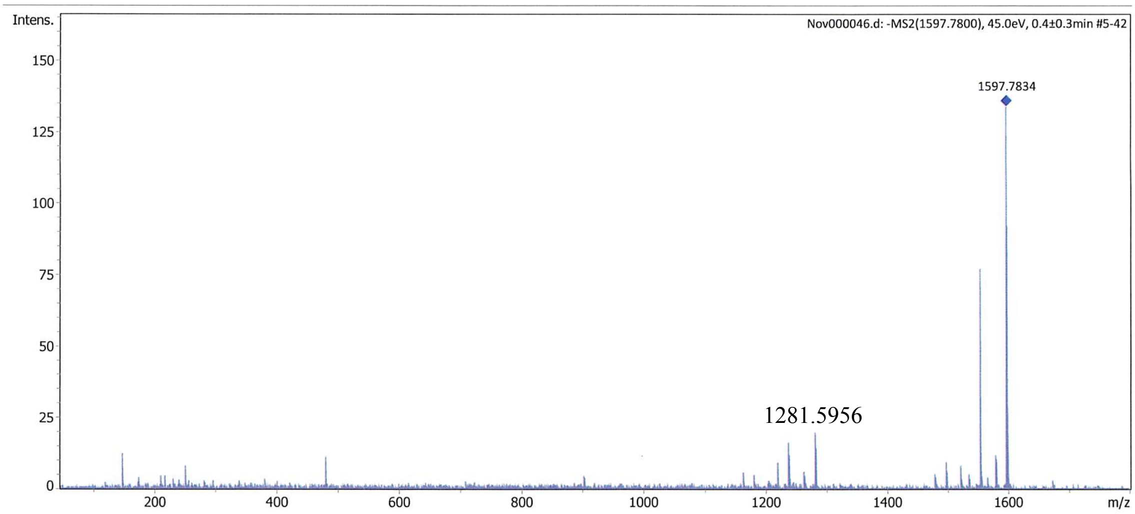

Figure S3: HR-negative-ion ESI-TOF-MS/MS of [M–H]⁻ ion of **1**

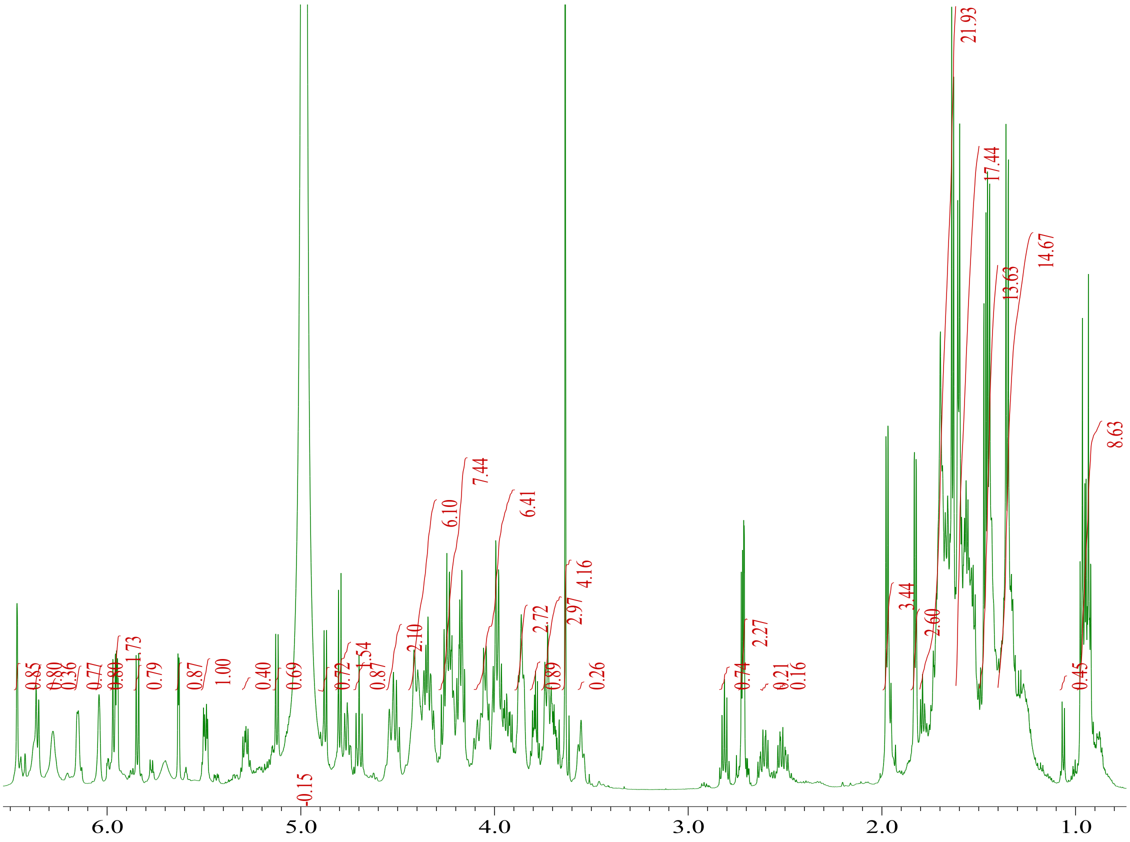

Figure S4: ^{1}H-NMR (600 MHz, pyridine-d_5) spectrum of **1**

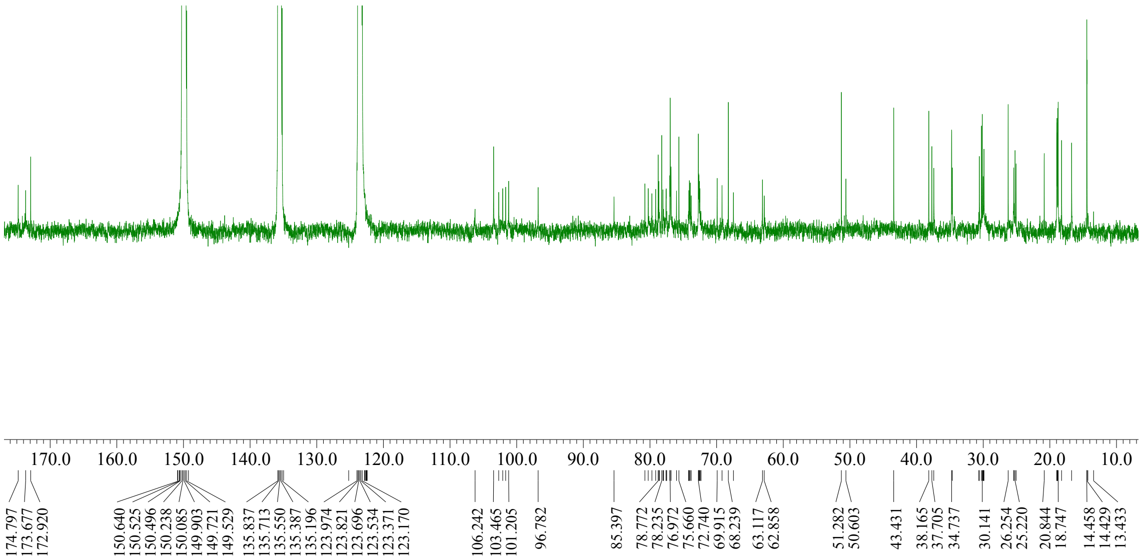

Figure S5: ^{13}C-NMR (150 MHz, pyridine-d_5) spectrum of **1**

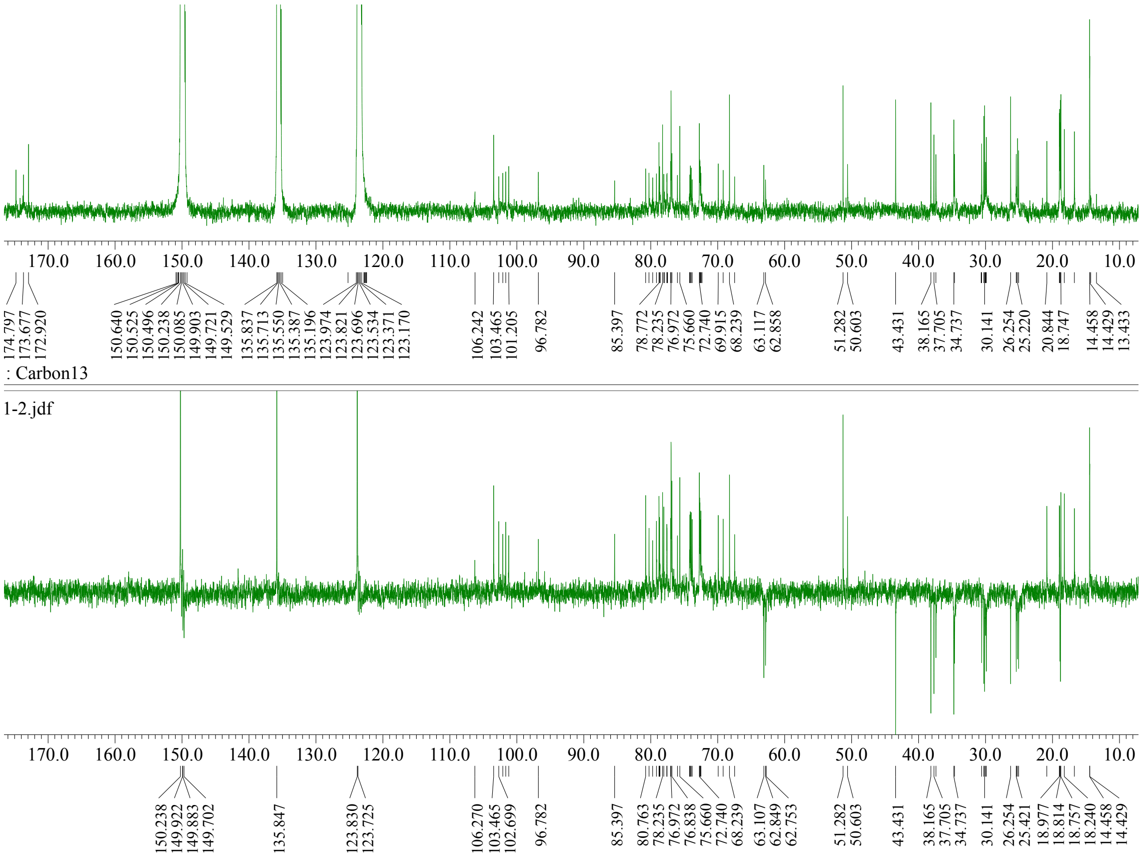

Figure S6: DEPT (150 MHz, pyridine-d_5) spectrum of **1**

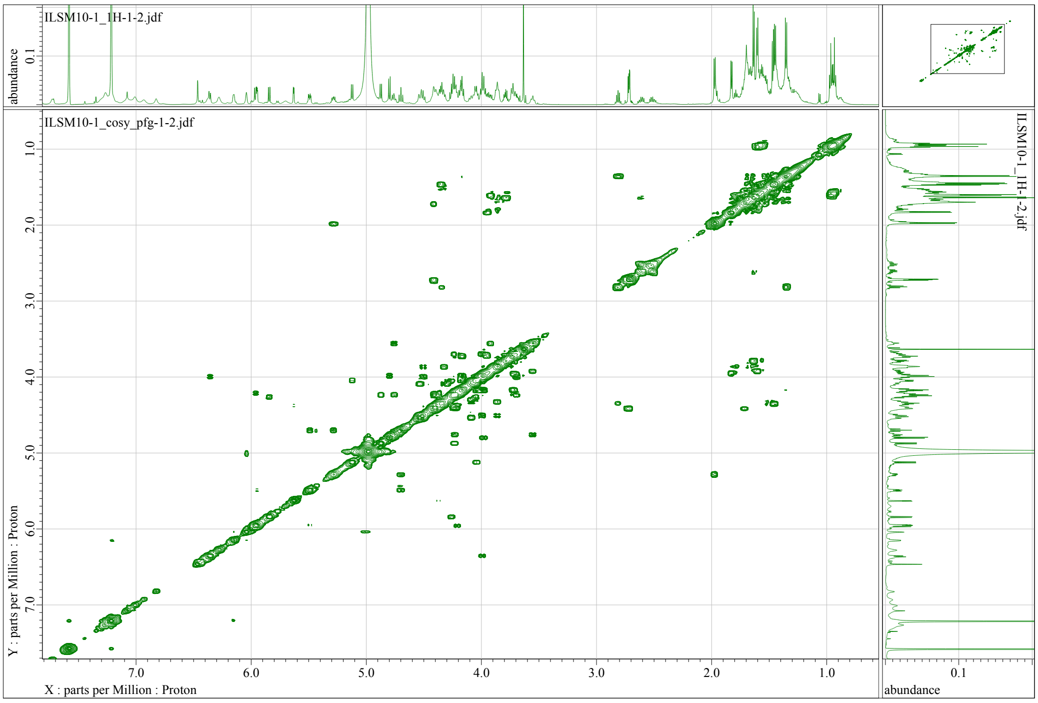

Figure S7: ^{1}H-^{1}H COSY (600 MHz, pyridine-d_5) spectrum of **1**

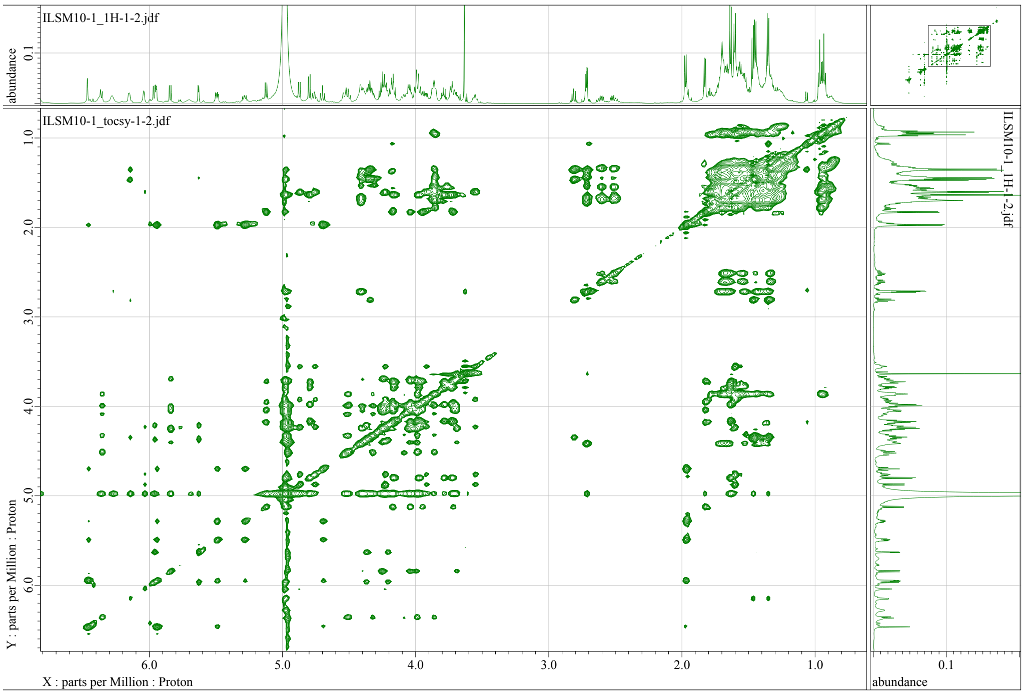

Figure S8: ^{1}H-^{1}H TOCSY (600 MHz, pyridine-d_5) spectrum of **1**

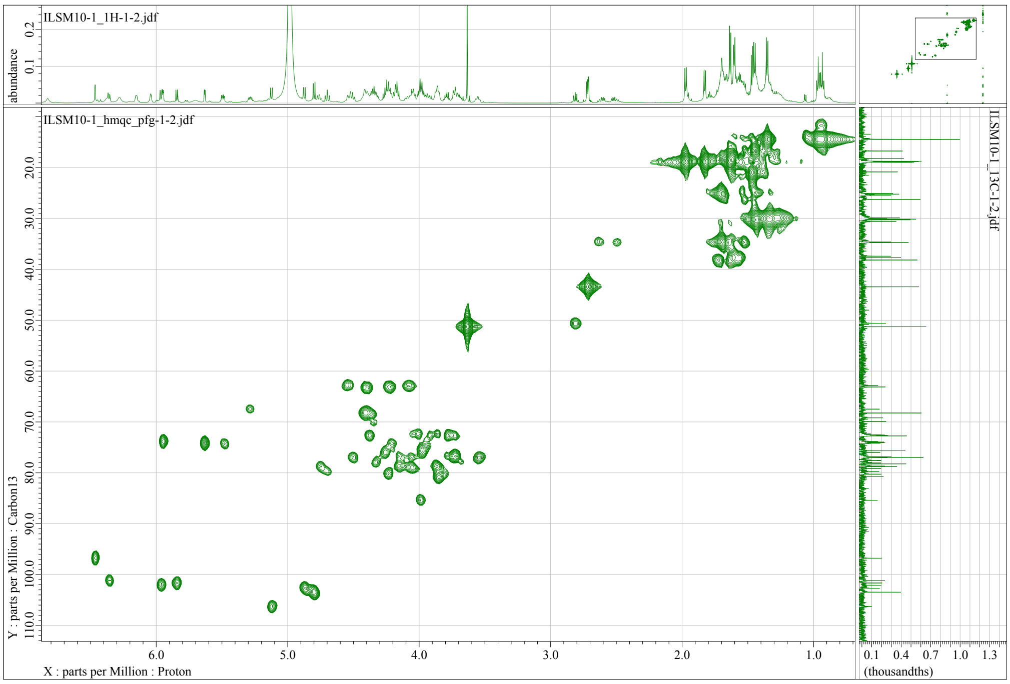

Figure S9: HMQC (600 MHz, pyridine-d_5) spectrum of **1**

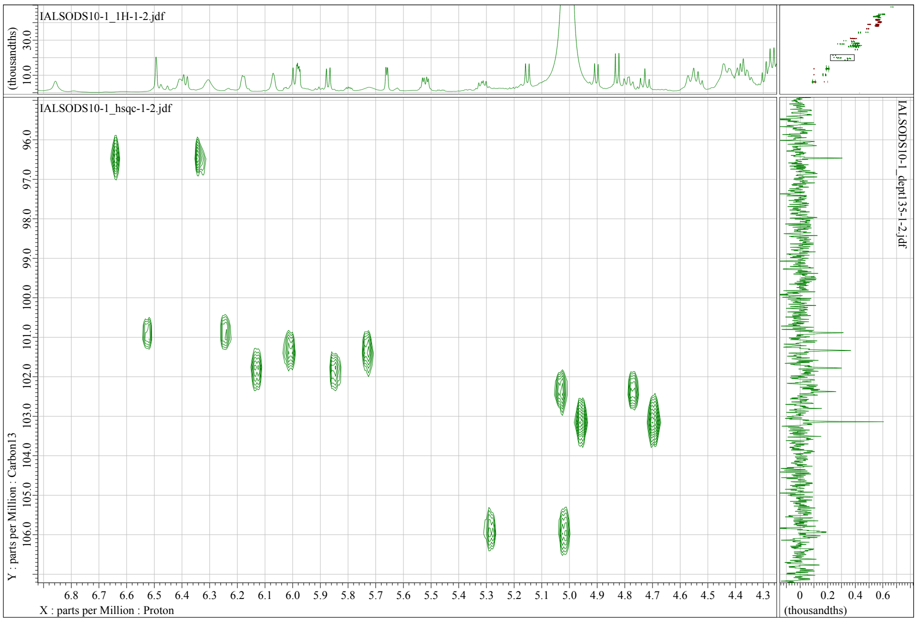

Figure S10: HSQC (600 MHz, pyridine-d_5) spectrum of **1**

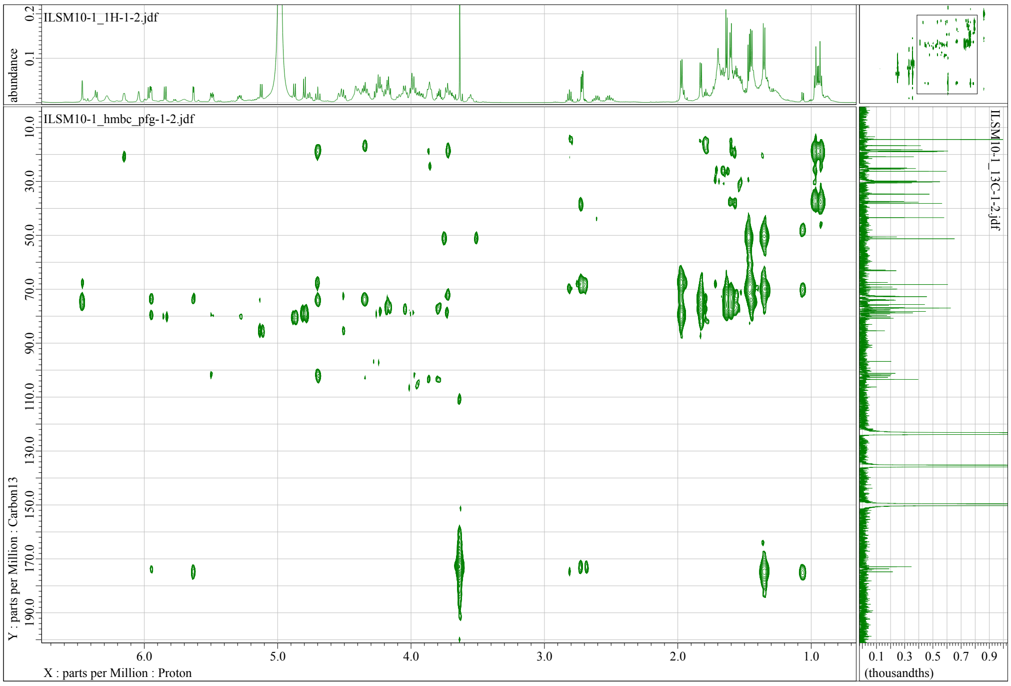

Figure S11: HMBC (600 MHz, pyridine-d_5) spectrum of **1**

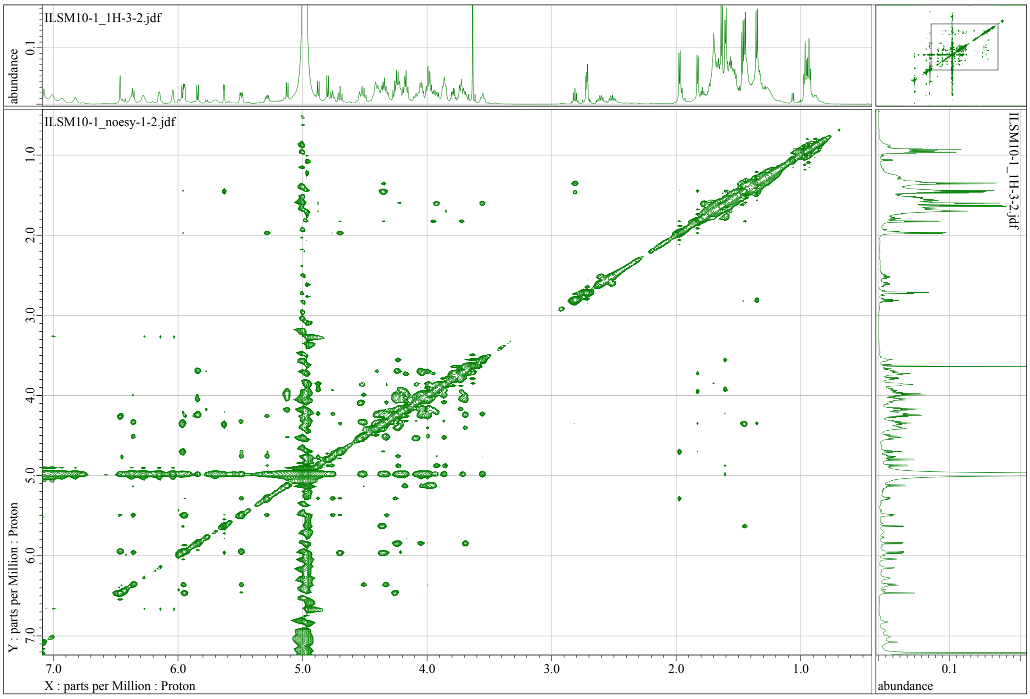

Figure S12: NOESY (600 MHz, pyridine-d_5) spectrum of **1**

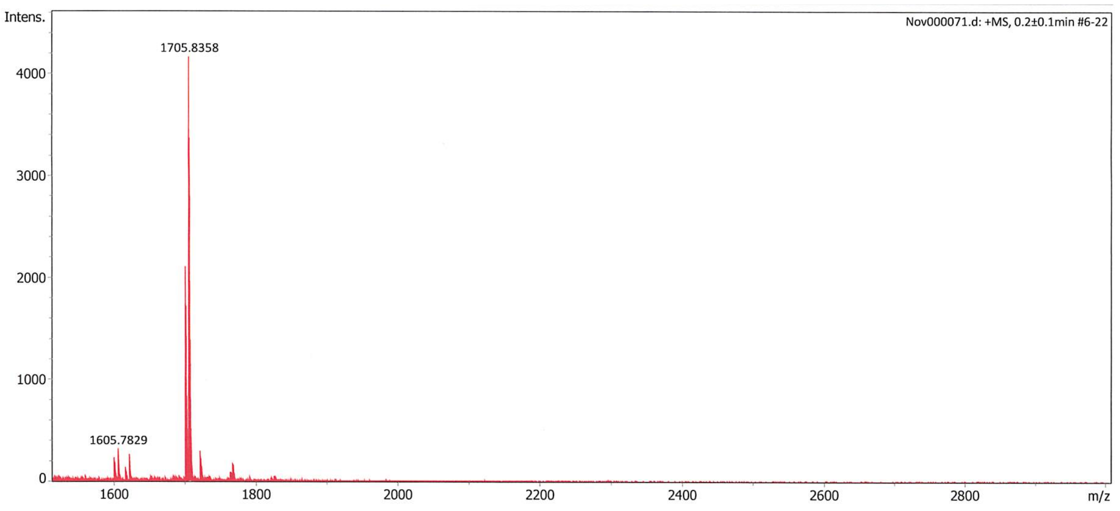

Figure S13. HR-positive-ion ESI-TOF-MS of **2**

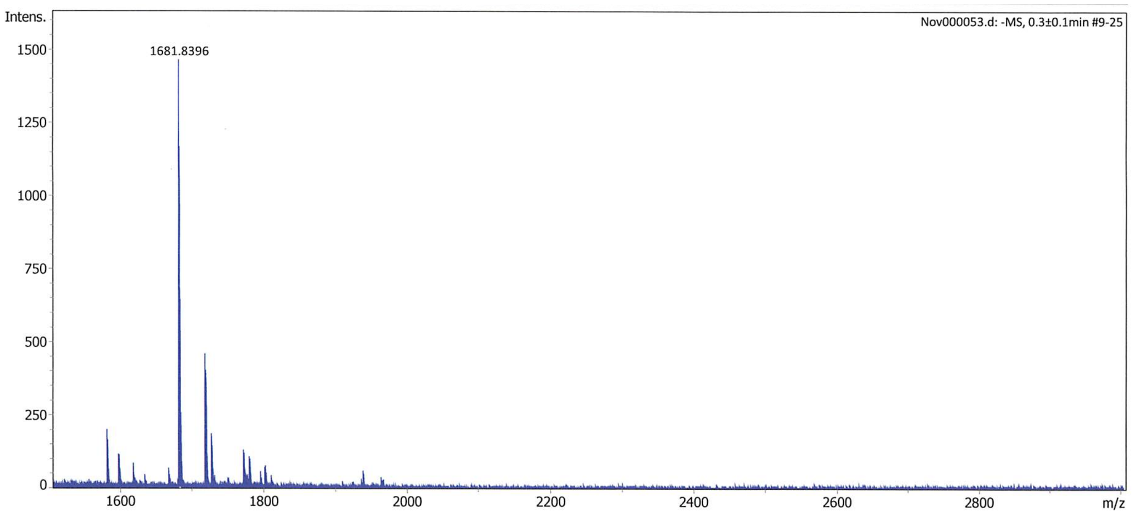

Figure S14: HR-negative-ion ESI-TOF-MS of **2**

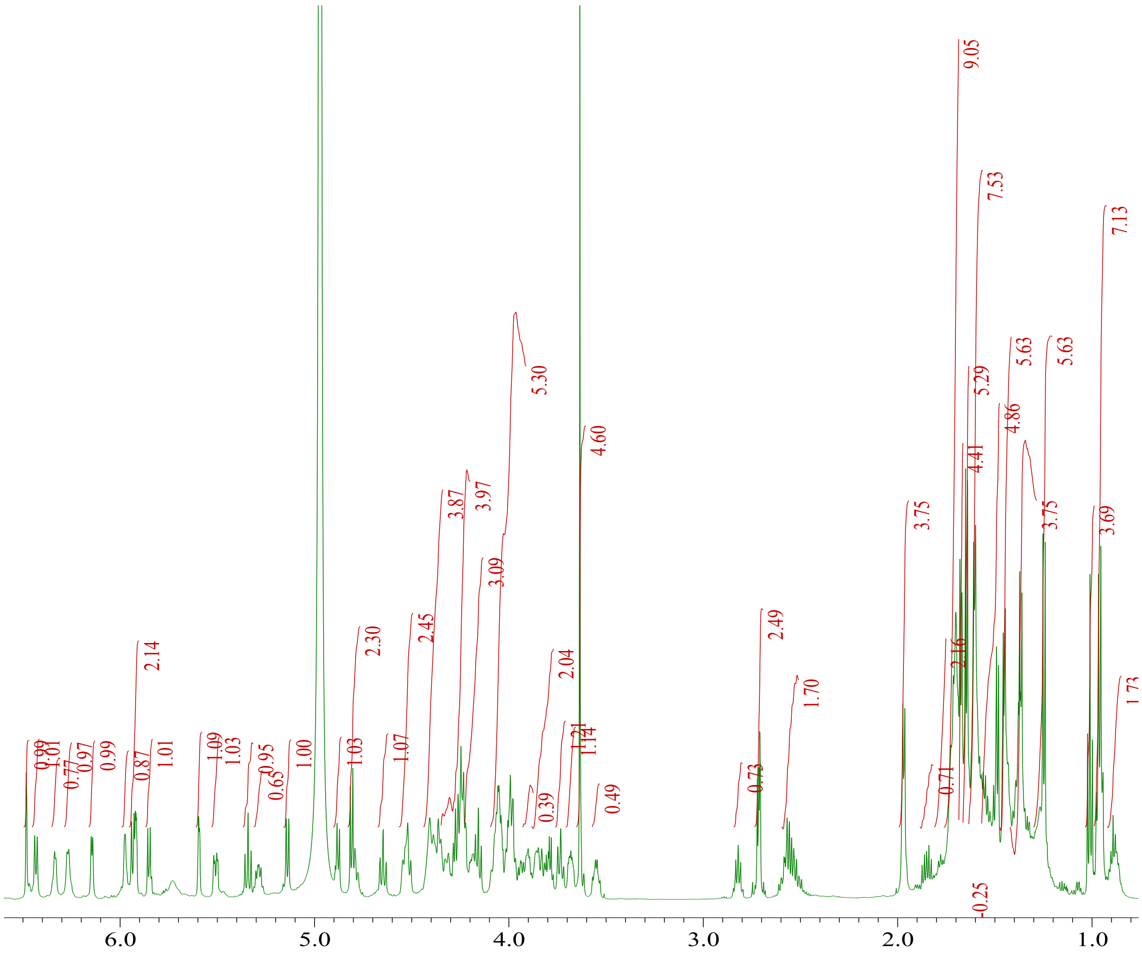

Figure S15: ^{1}H-NMR (600 MHz, pyridine-d_5) spectrum of **2**

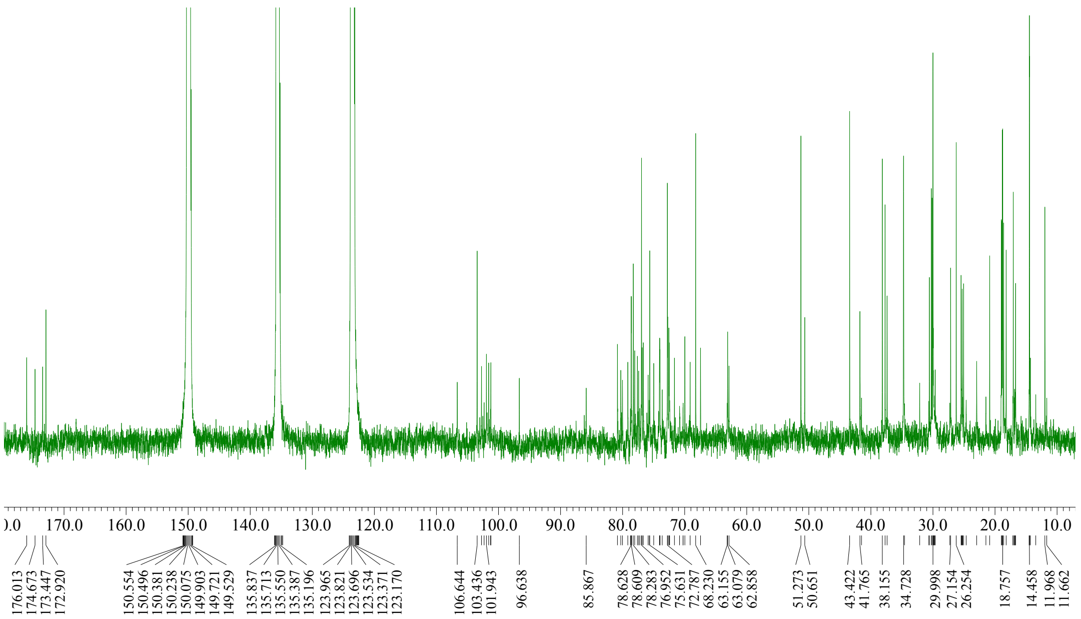

Figure S16: ^{13}C-NMR (150 MHz, pyridine-d_5) spectrum of **2**

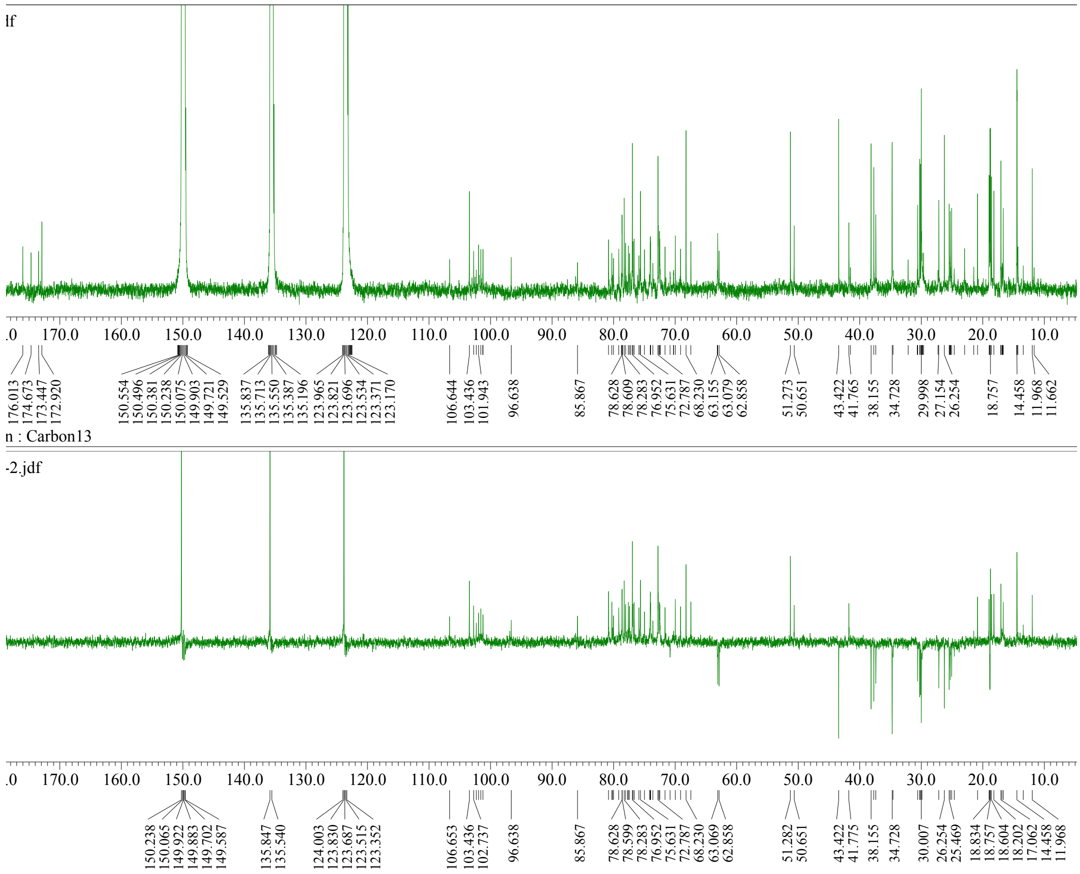

Figure S17: DEPT (150 MHz, pyridine-d_5) spectrum of **2**

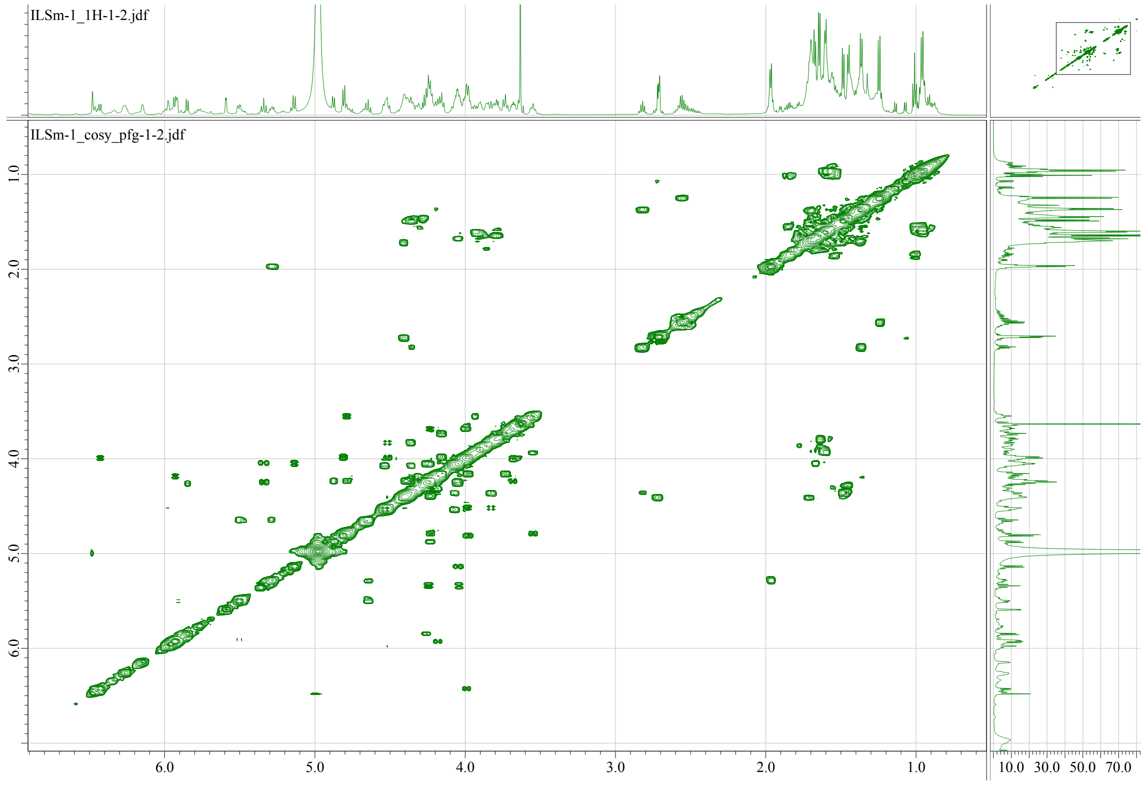

Figure S18: ^{1}H-^{1}H COSY (600 MHz, pyridine-d_5) spectrum of **2**

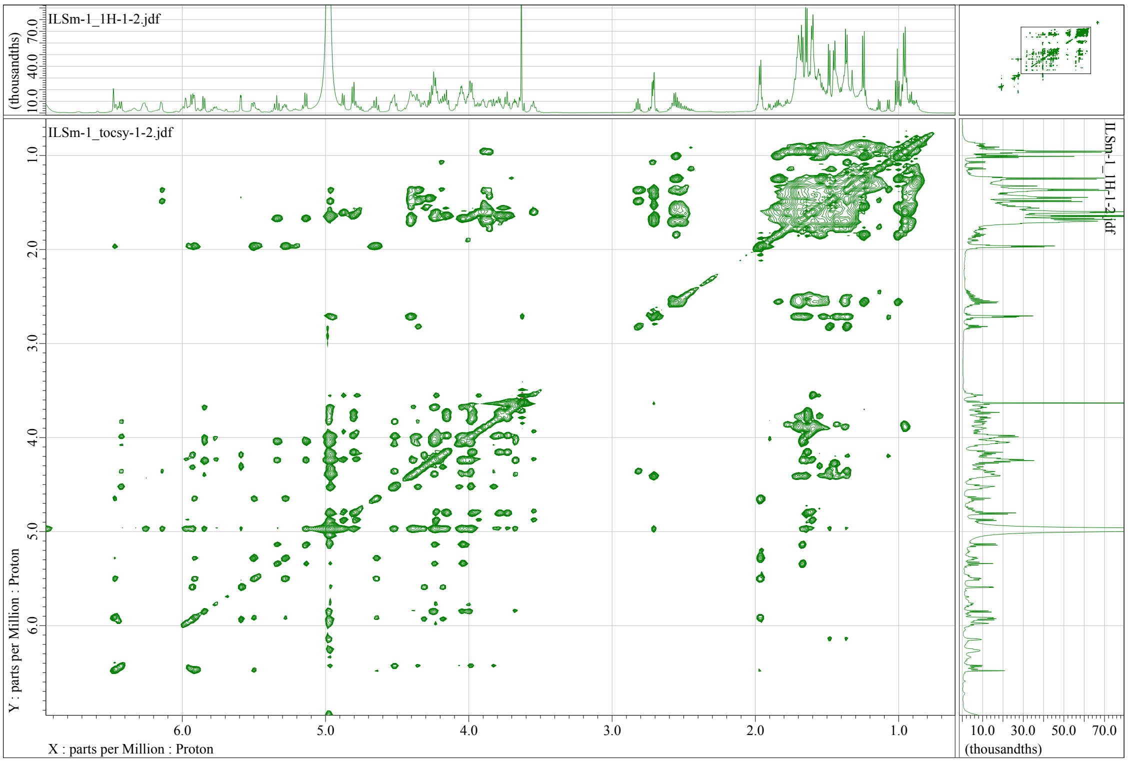

Figure S19: ^{1}H-^{1}H TOCSY (600 MHz, pyridine-d_5) spectrum of **2**

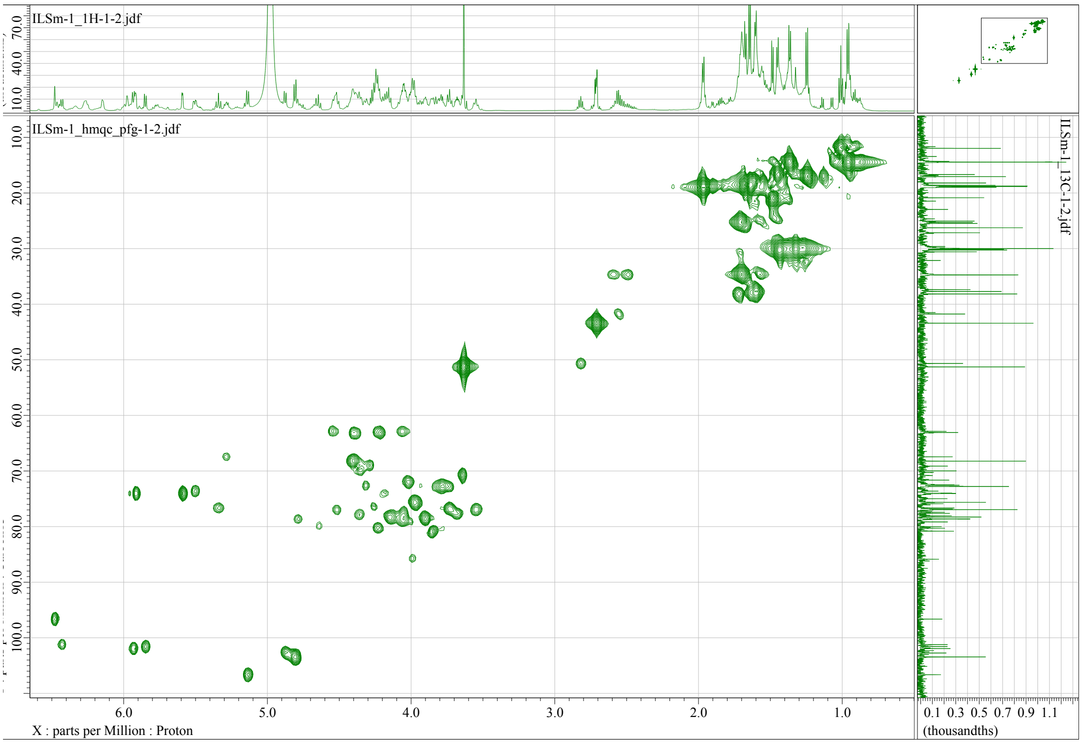

Figure S20: HMQC (600 MHz, pyridine-d_5) spectrum of **2**

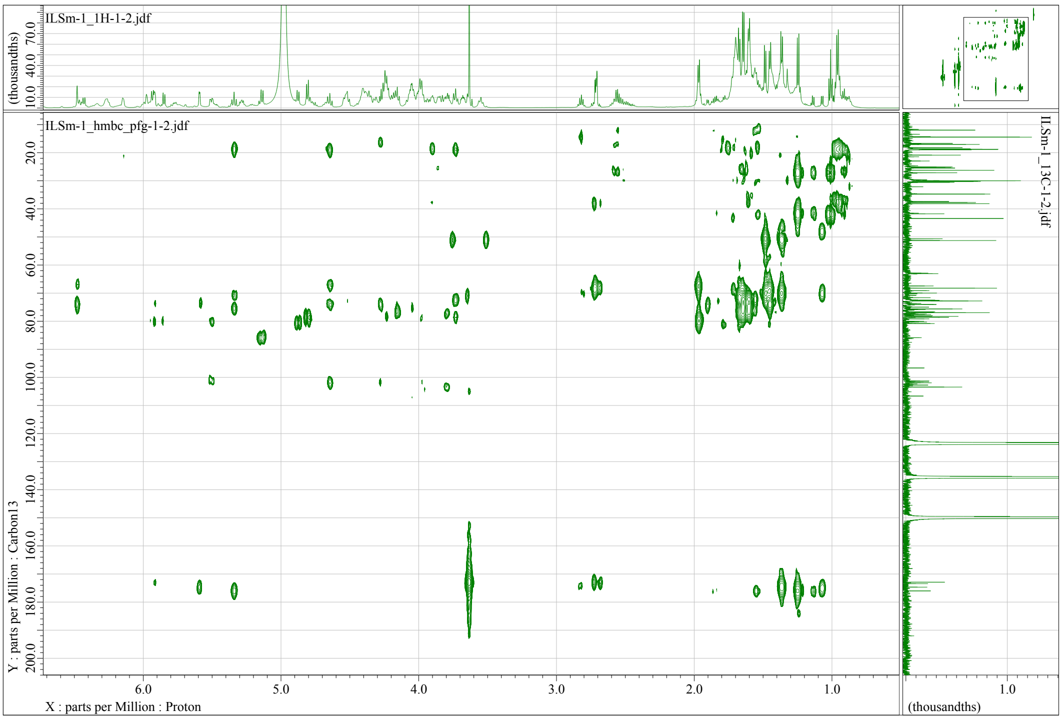

Figure S21: HMBC (600 MHz, pyridine-d_5) spectrum of **2**

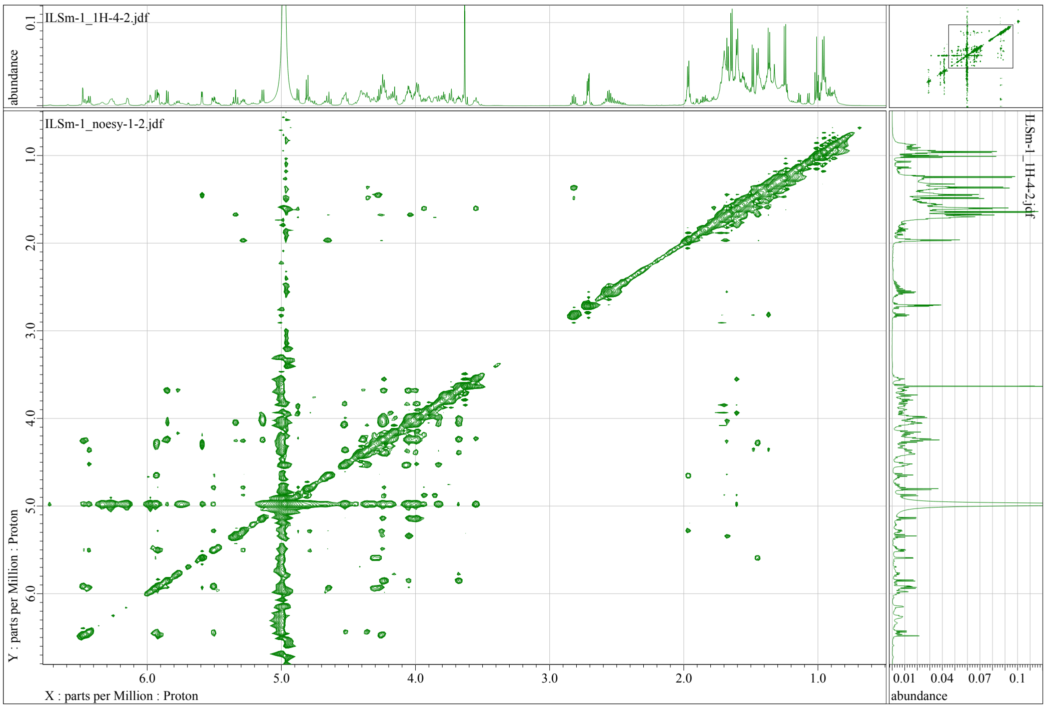

Figure S22: NOESY (600 MHz, pyridine-d_5) spectrum of **1**

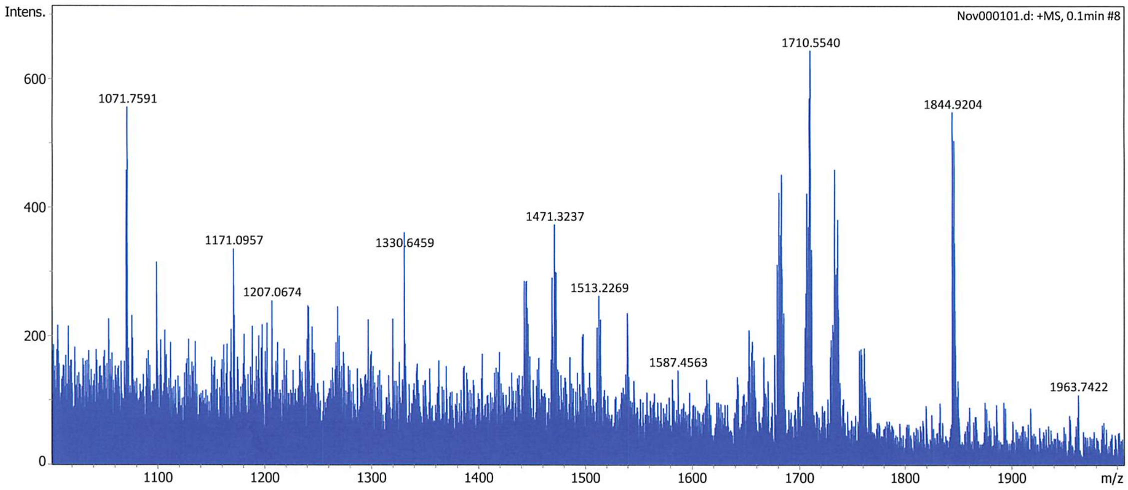

Figure S23: HR-positive-ion ESI-TOF-MS (+$HCOONH_4$) of **3**

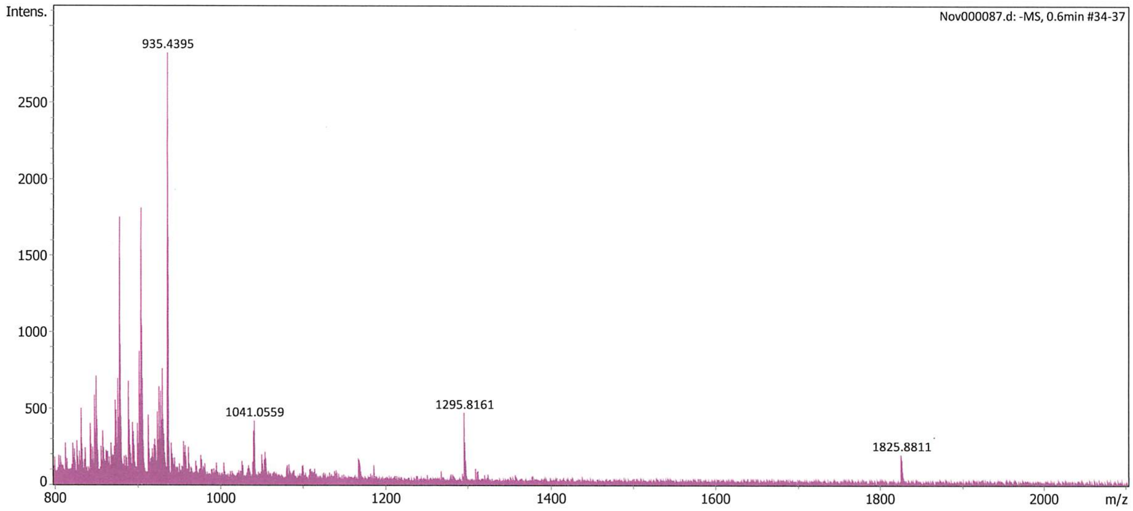

Figure S24: HR-negative-ion ESI-TOF-MS of **3**

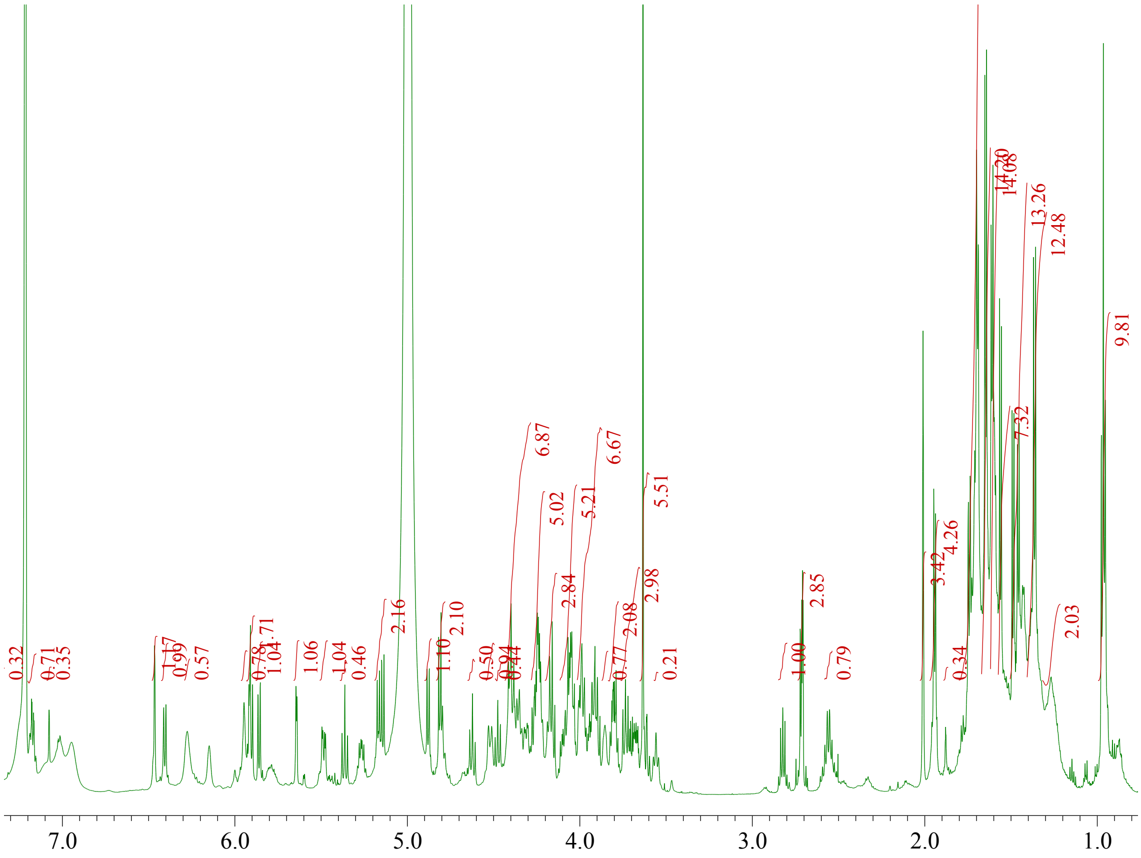

Figure S25: ^{1}H-NMR (600 MHz, pyridine-d_5) spectrum of **3**

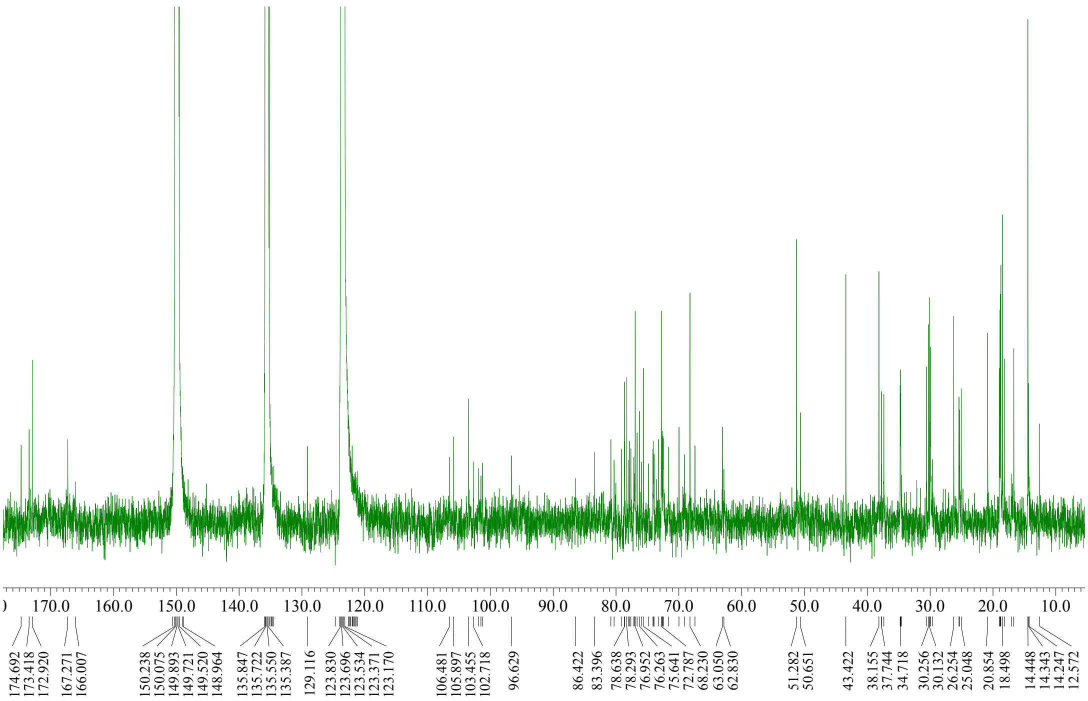

Figure S26: ^{13}C-NMR (150 MHz, pyridine-d_5) spectrum of **3**

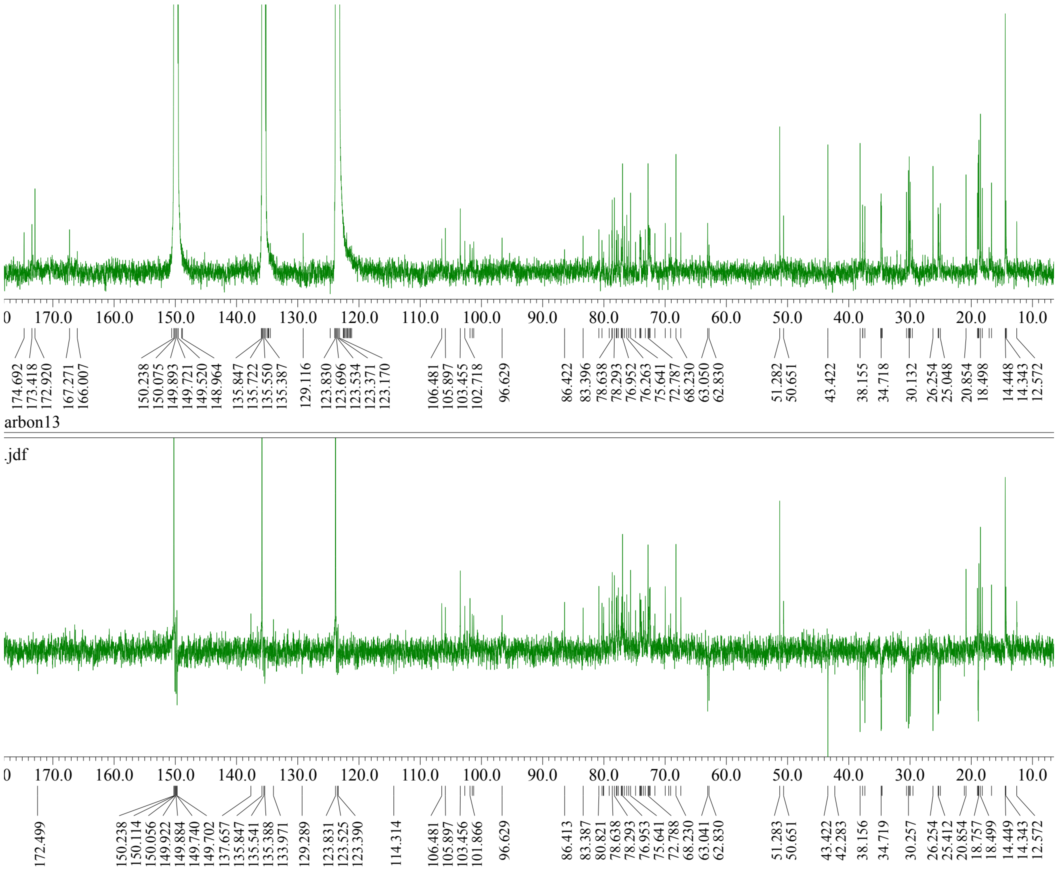

Figure S27: DEPT (150 MHz, pyridine-d_5) spectrum of **3**

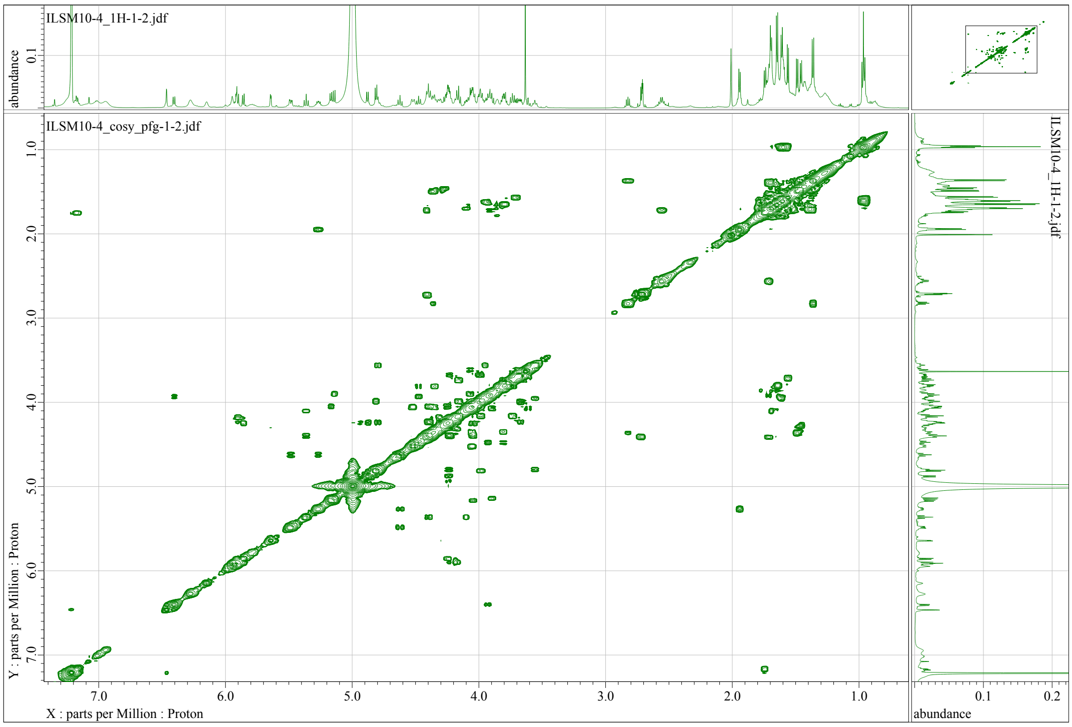

Figure S28: ^{1}H-^{1}H COSY (600 MHz, pyridine-d_5) spectrum of **3**

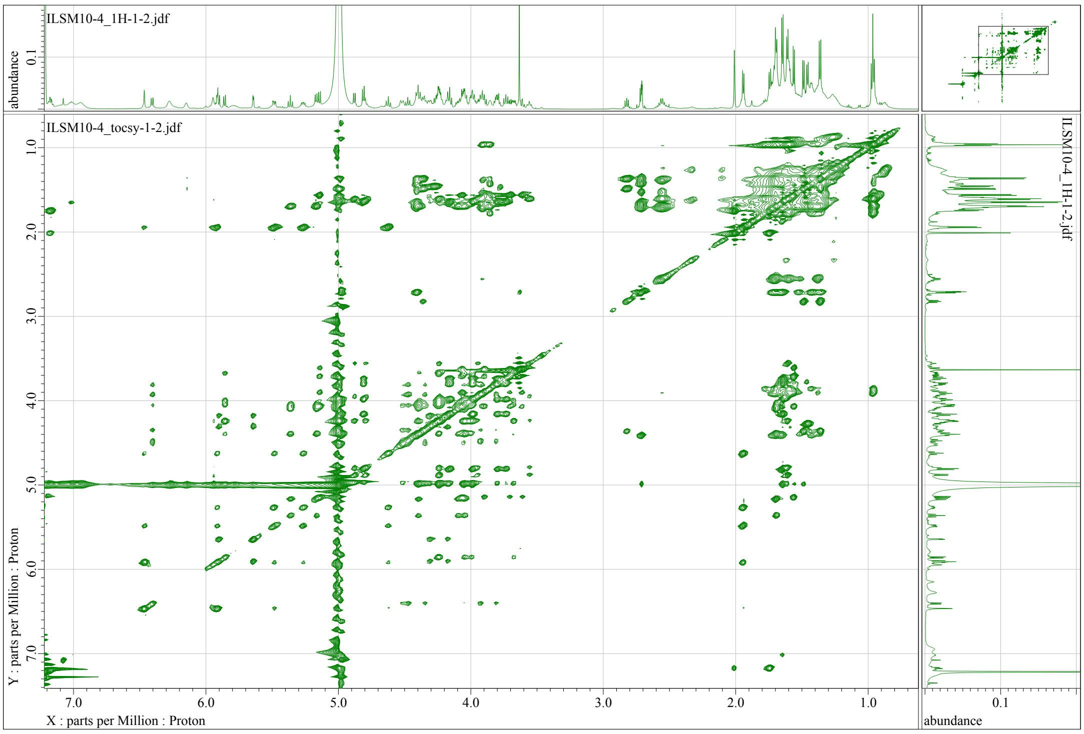

Figure S29: ^{1}H-^{1}H TOCSY (600 MHz, pyridine-d_5) spectrum of **3**

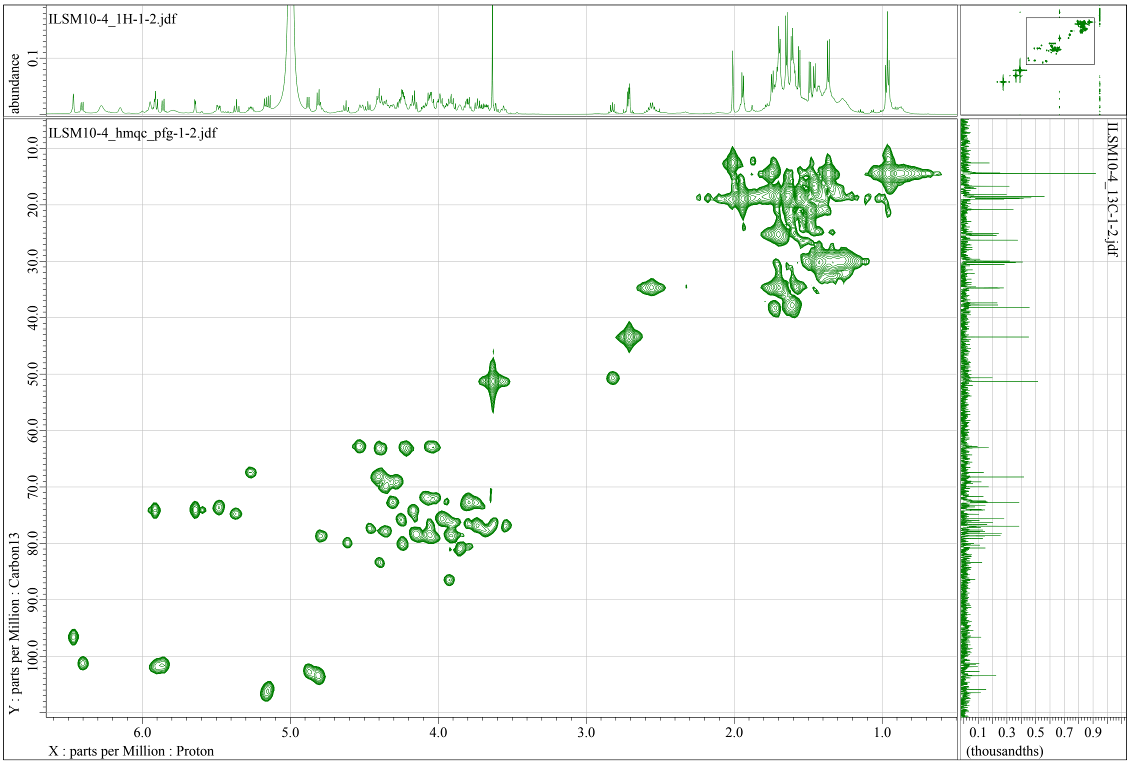

Figure S30: HMQC (600 MHz, pyridine-d_5) spectrum of **3**

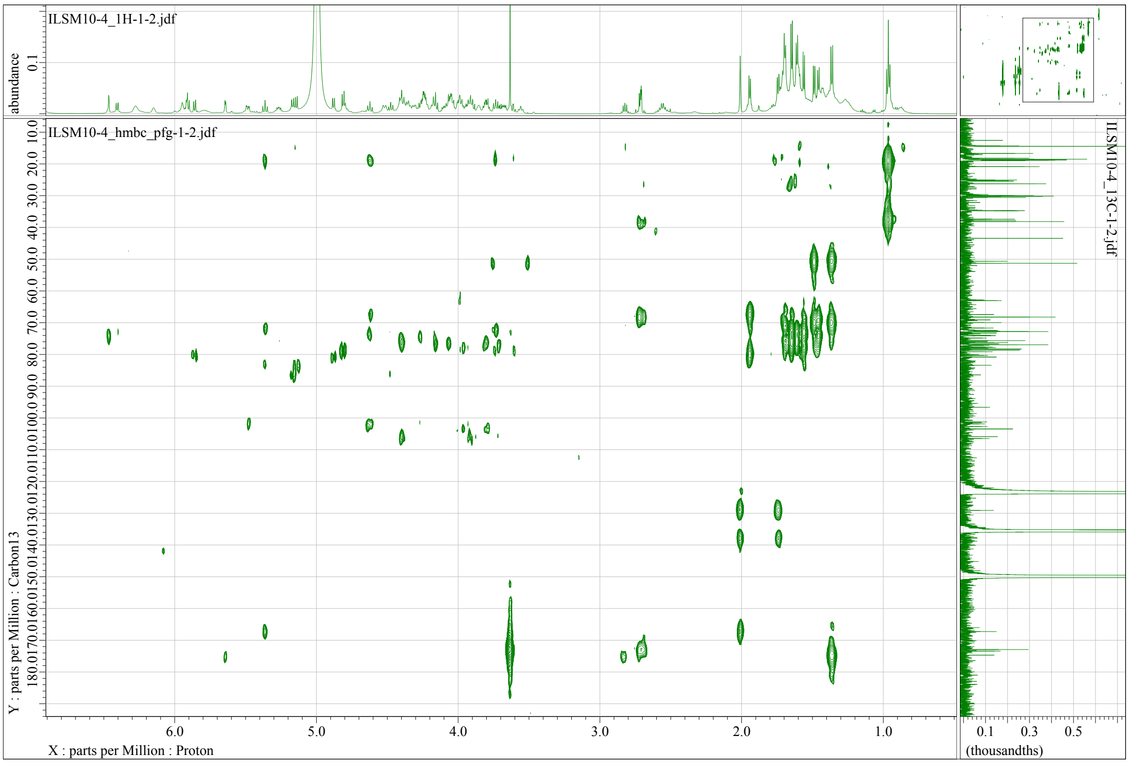

Figure S31: HMBC (600 MHz, pyridine-d_5) spectrum of **3**

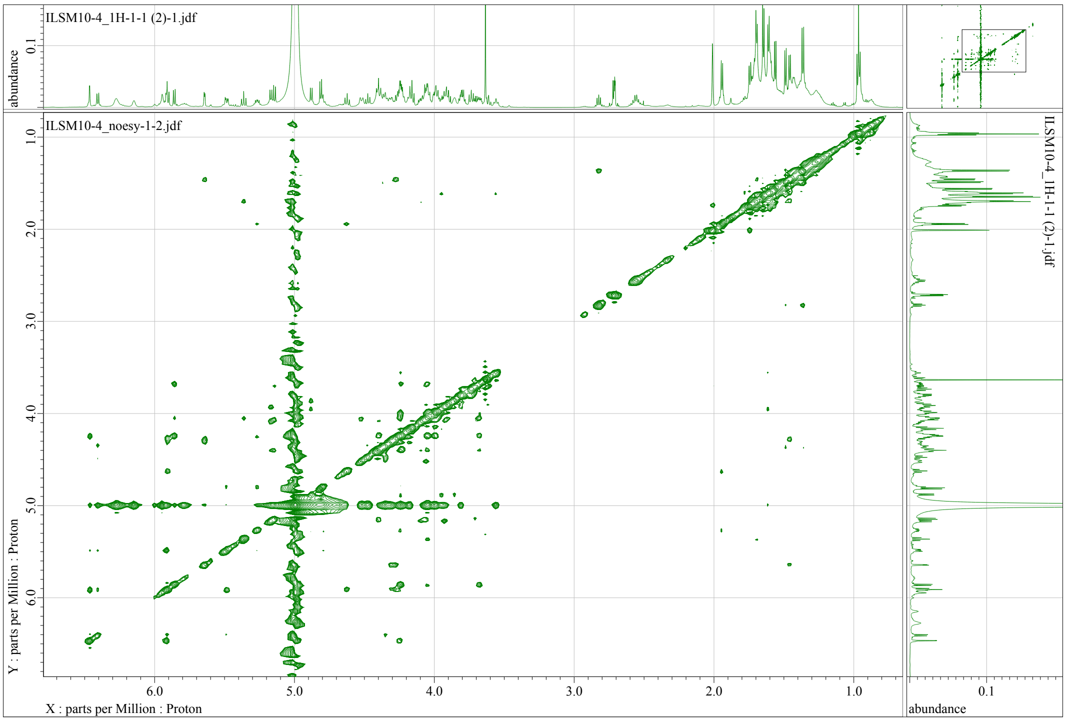

Figure S32: NOESY (600 MHz, pyridine-d_5) spectrum of **3**

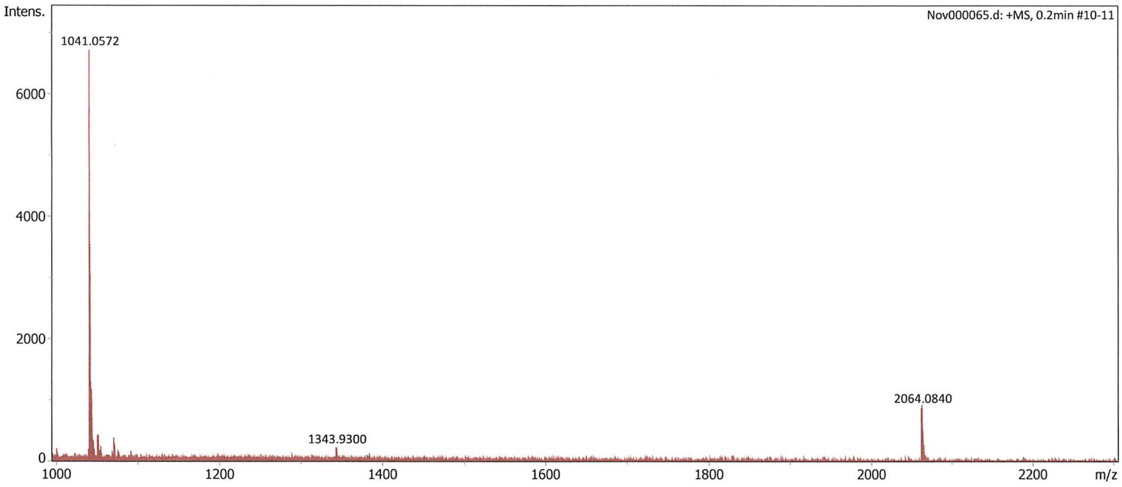

Figure S33: HR-positive-ion ESI-TOF-MS (+$HCOONH_4$) of **4**

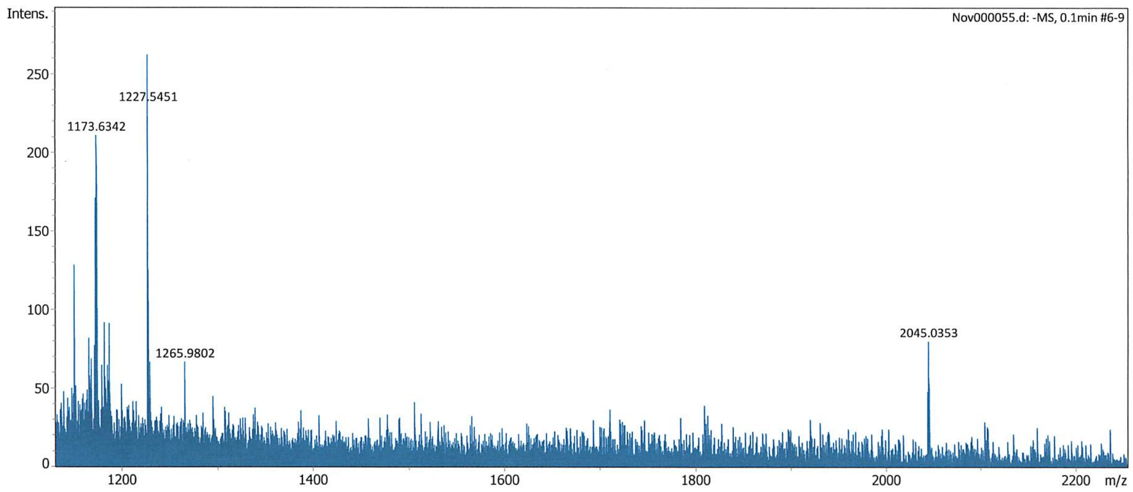

Figure S34: HR-negative-ion ESI-TOF-MS of **4**

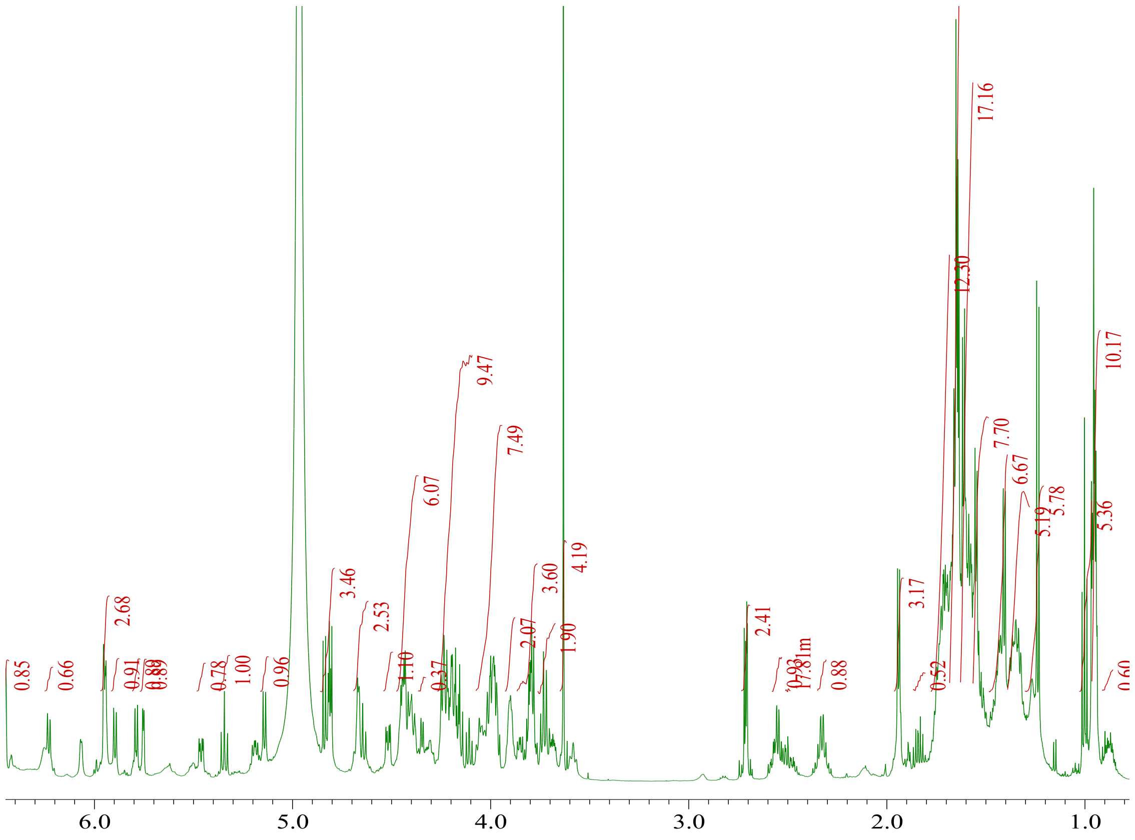

Figure S35: ^{1}H-NMR (600 MHz, pyridine-d_5) spectrum of **4**

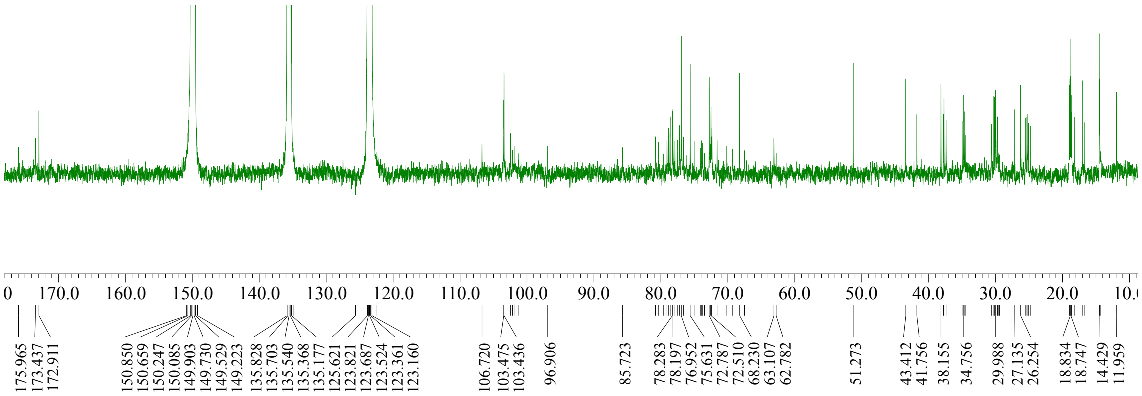

Figure S36: ^{13}C-NMR (150 MHz, pyridine-d_5) spectrum of **4**

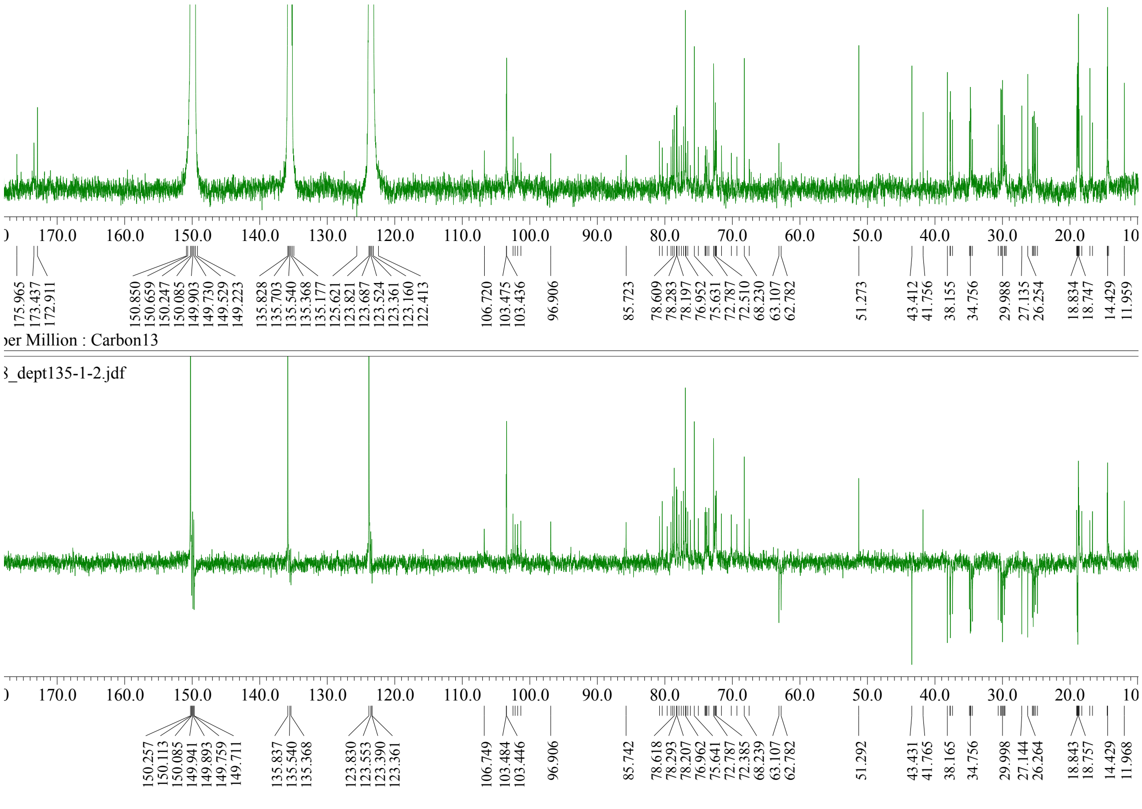

Figure S37: DEPT (150 MHz, pyridine-d_5) spectrum of **4**

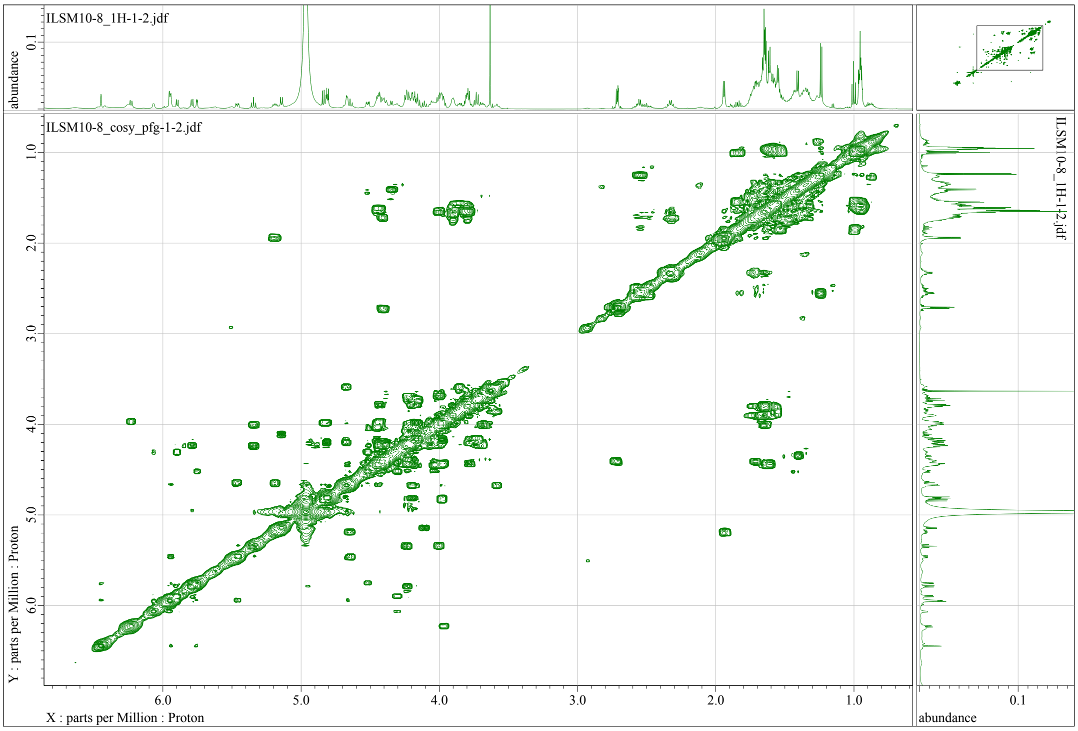

Figure S38: ^{1}H-^{1}H COSY (600 MHz, pyridine-d_5) spectrum of **4**

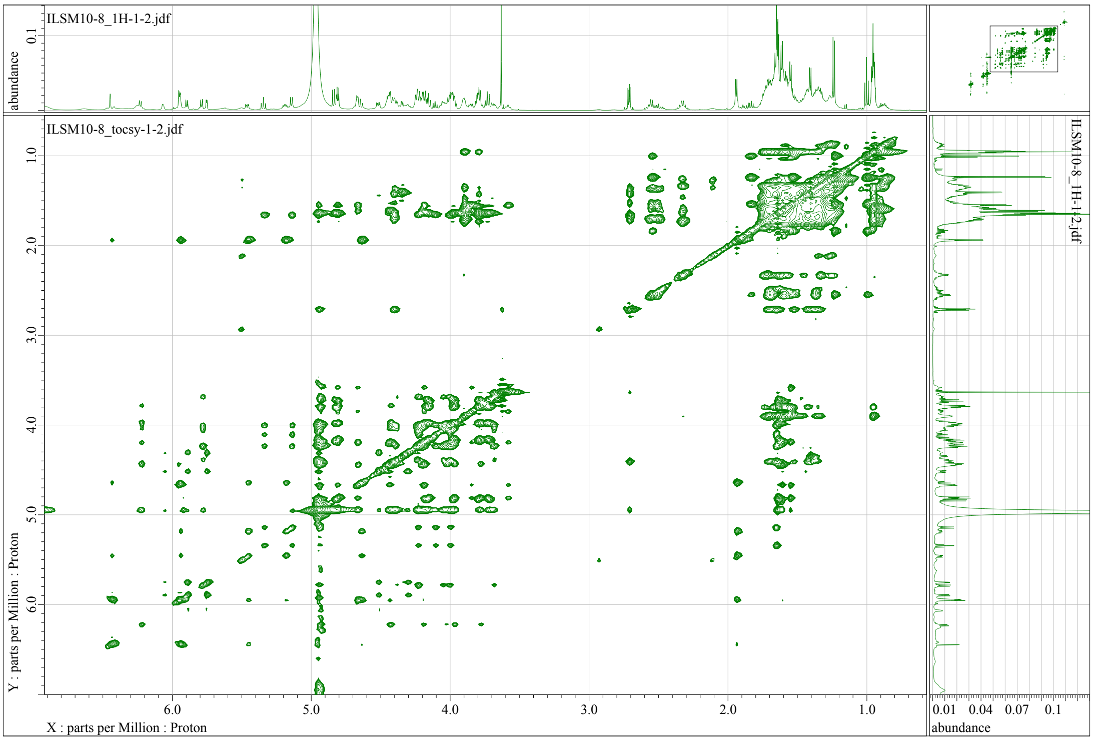

Figure S39: ^{1}H-^{1}H TOCSY (600 MHz, pyridine-d_5) spectrum of **4**

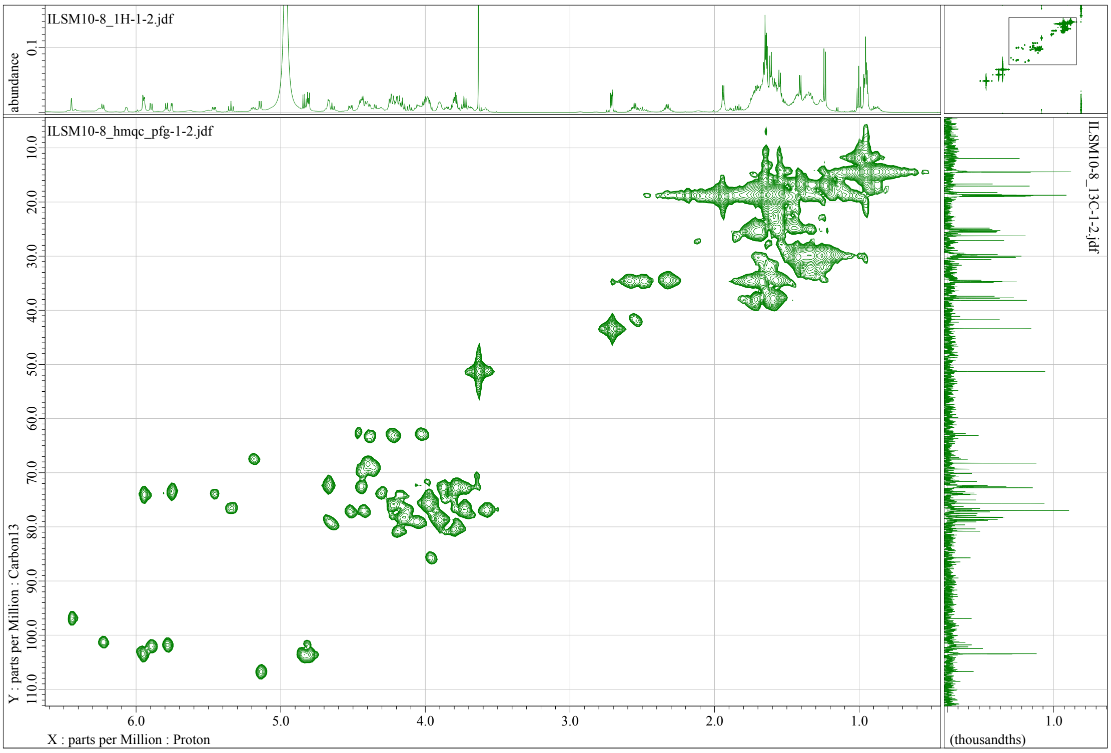

Figure S40: HMQC (600 MHz, pyridine-d_5) spectrum of **4**

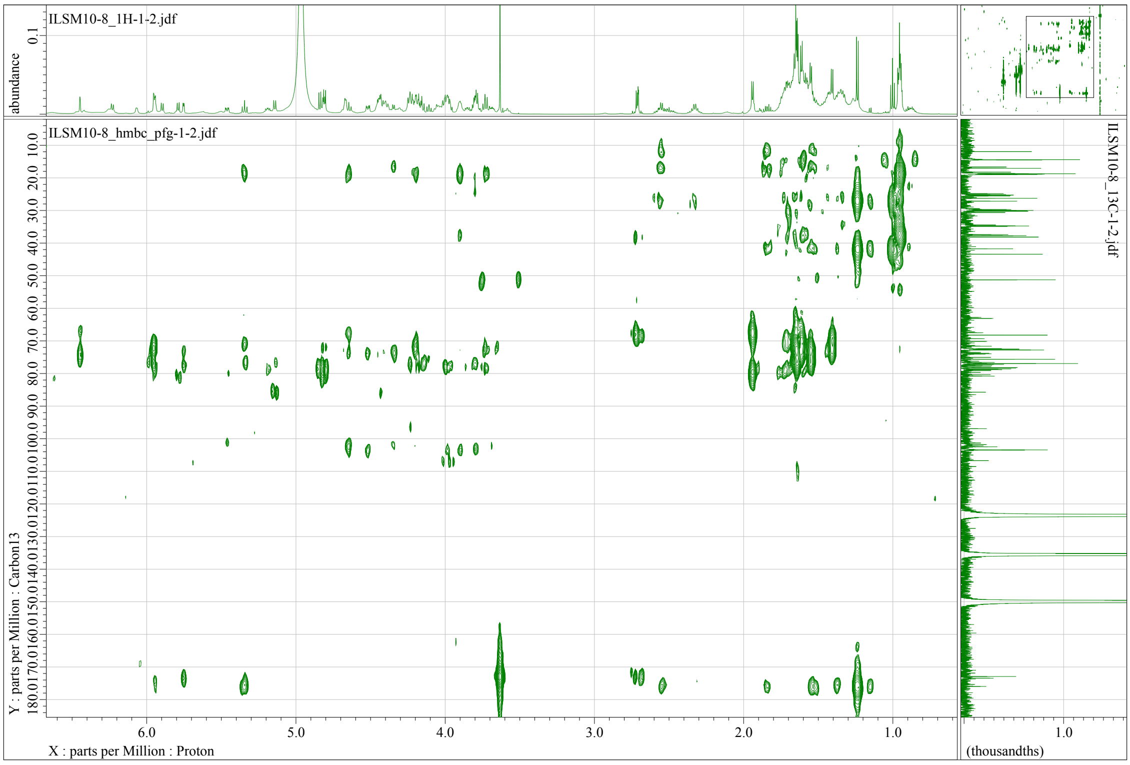

Figure S41: HMBC (600 MHz, pyridine-d_5) spectrum of **4**

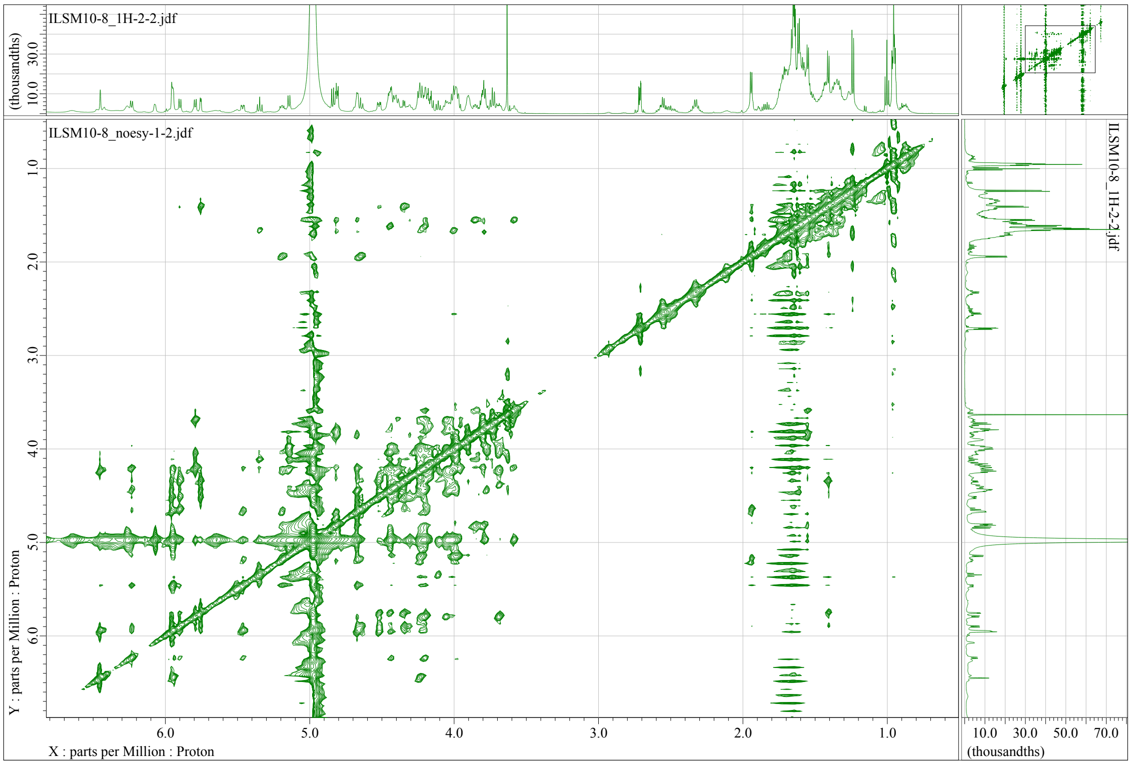

Figure S42: NOESY (600 MHz, pyridine-d_5) spectrum of **4**

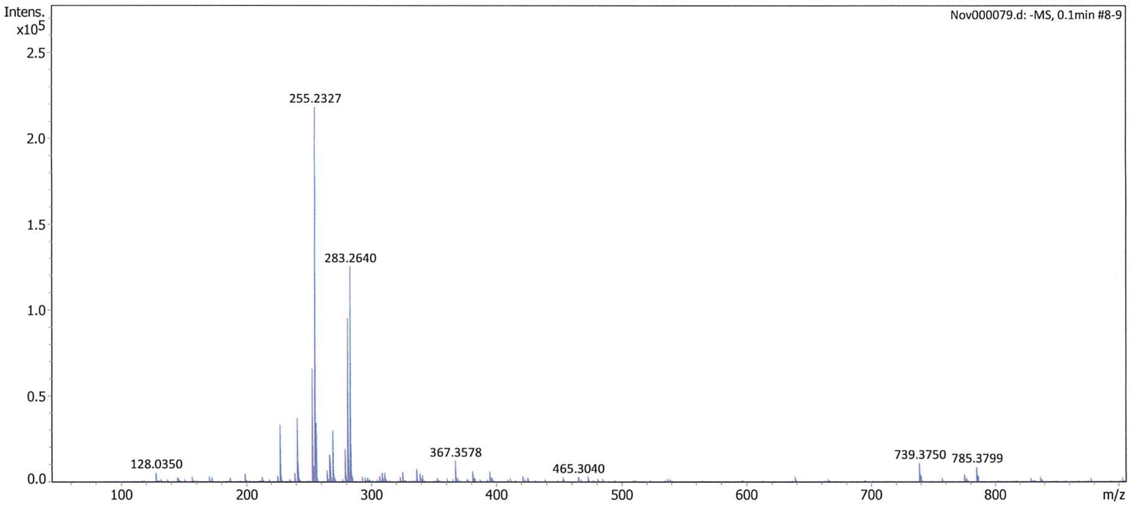

Figure S43: HR-negative-ion ESI-TOF-MS of **5**

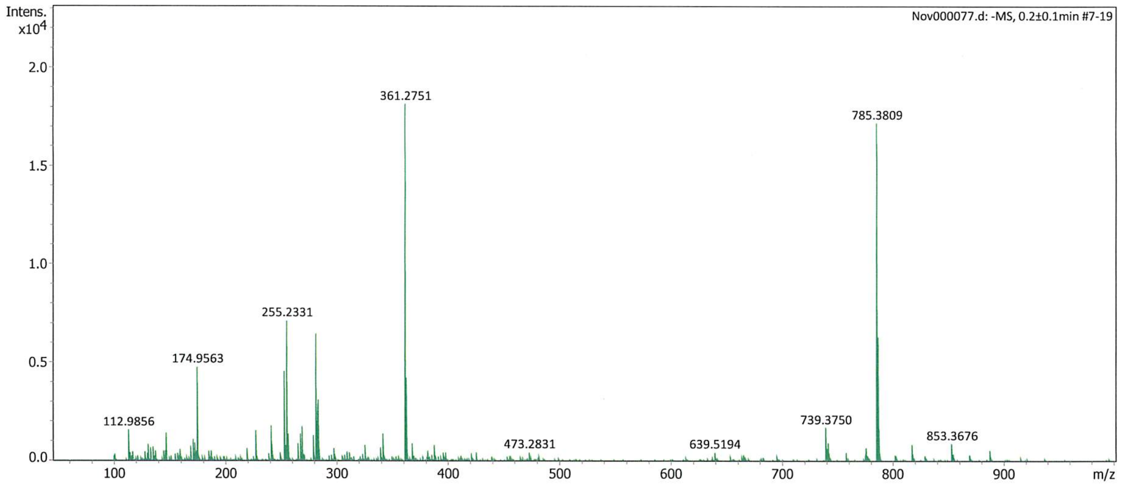

Figure S44: HR-negative-ion ESI-TOF-MS (+$HCOONH_4$) of **5**

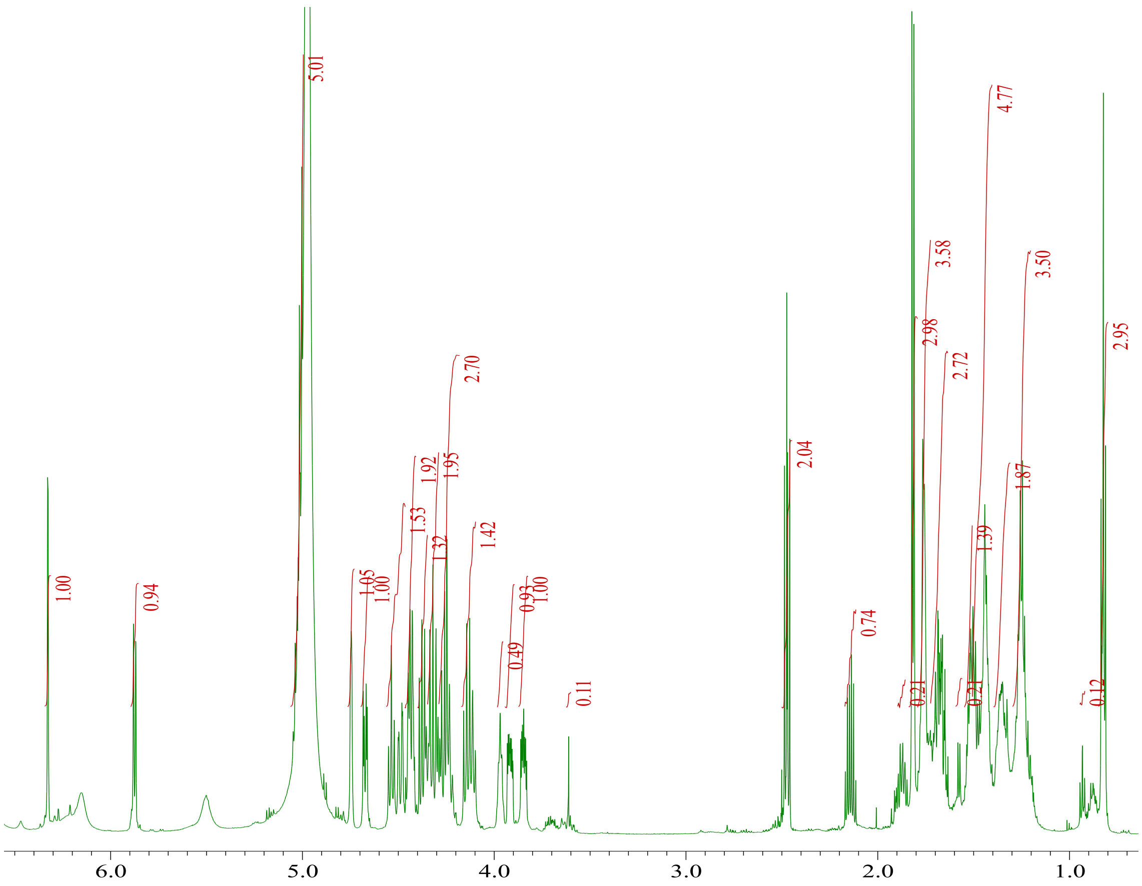

Figure S45: ^{1}H-NMR (600 MHz, pyridine-d_5) spectrum of **5**

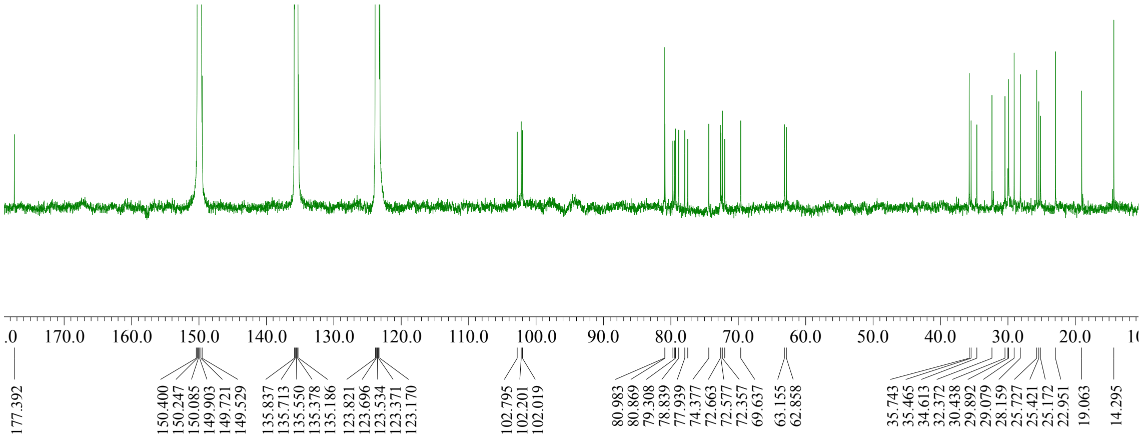

Figure S46: ^{13}C-NMR (150 MHz, pyridine-d_5) spectrum of **5**

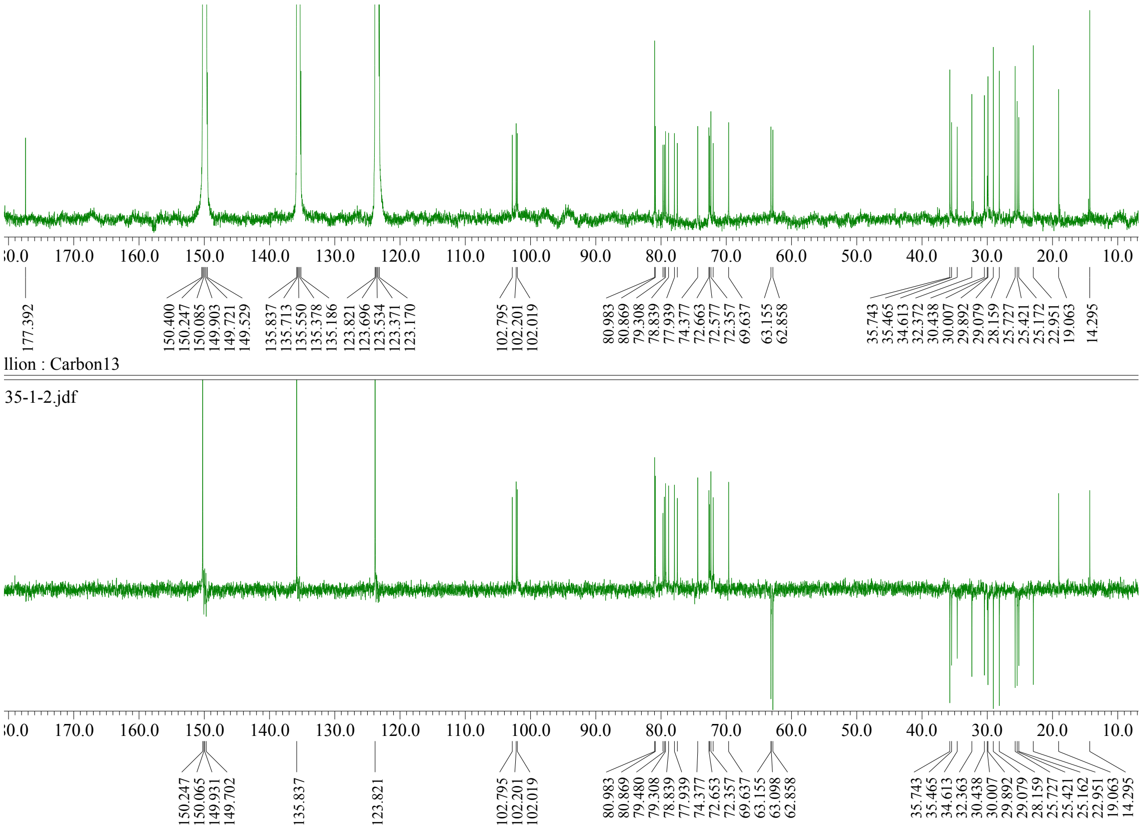

Figure S47: DEPT (150 MHz, pyridine-d_5) spectrum of **5**

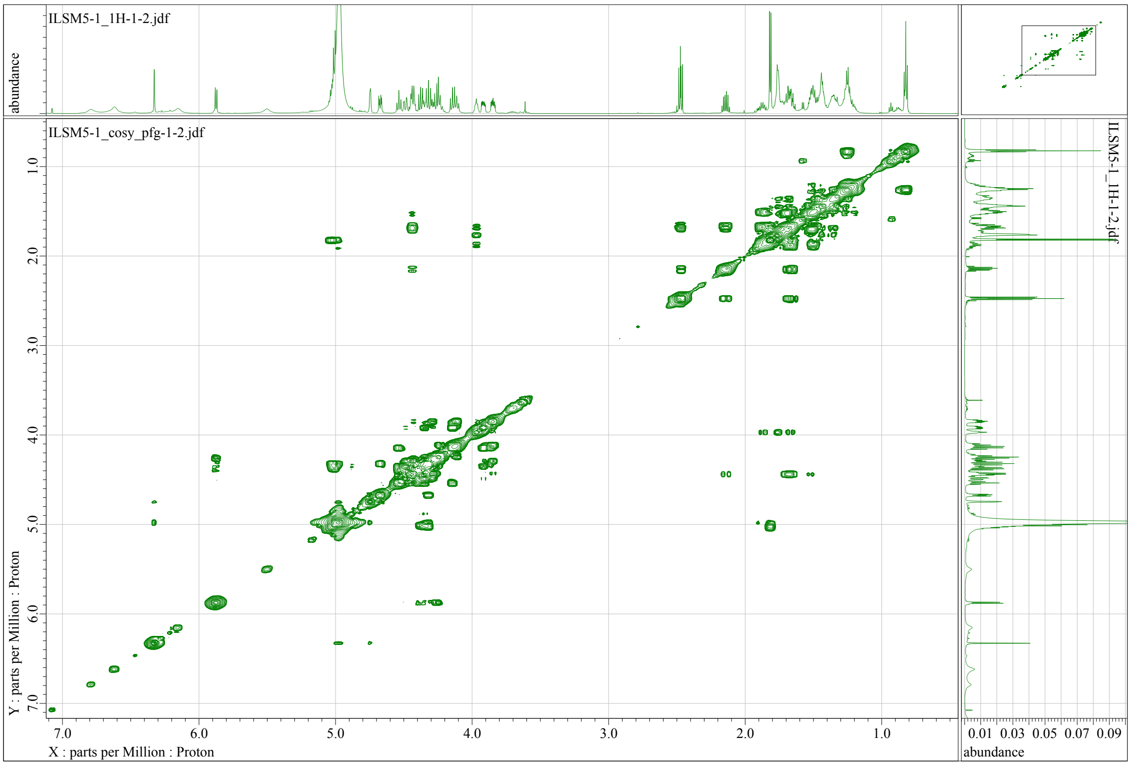

Figure S48: ^{1}H-^{1}H COSY (600 MHz, pyridine-d_5) spectrum of **5**

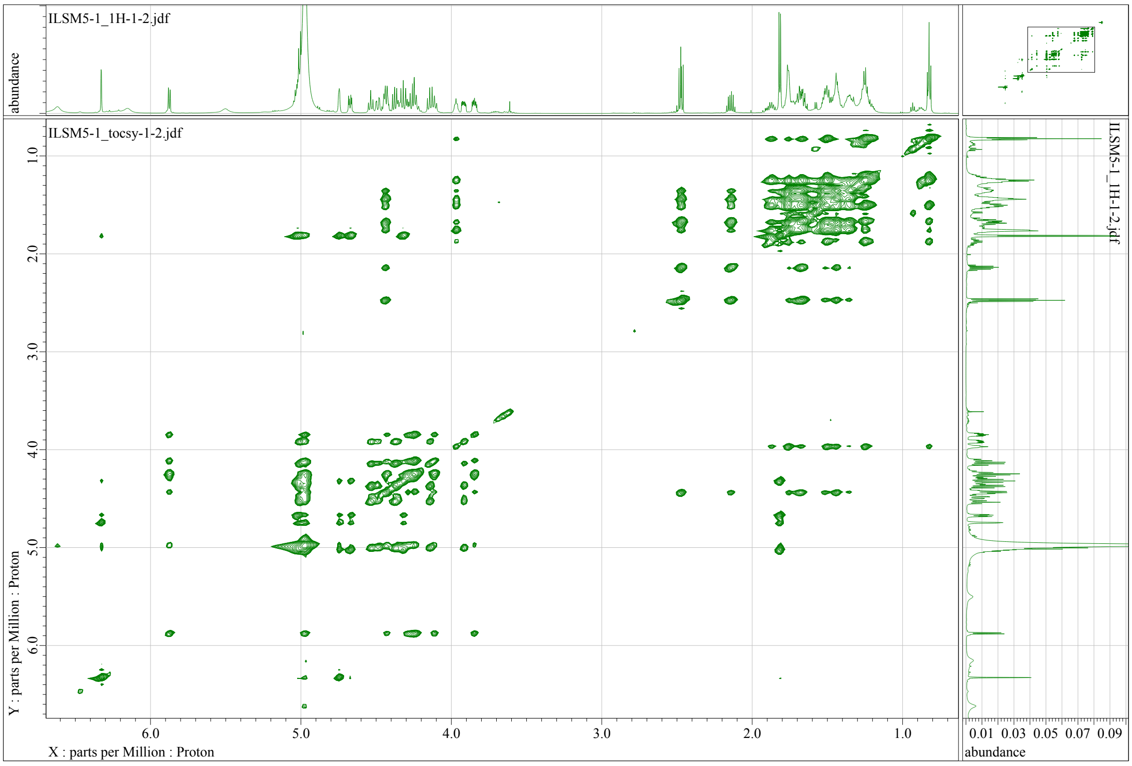

Figure S49: ^{1}H-^{1}H TOCSY (600 MHz, pyridine-d_5) spectrum of **5**

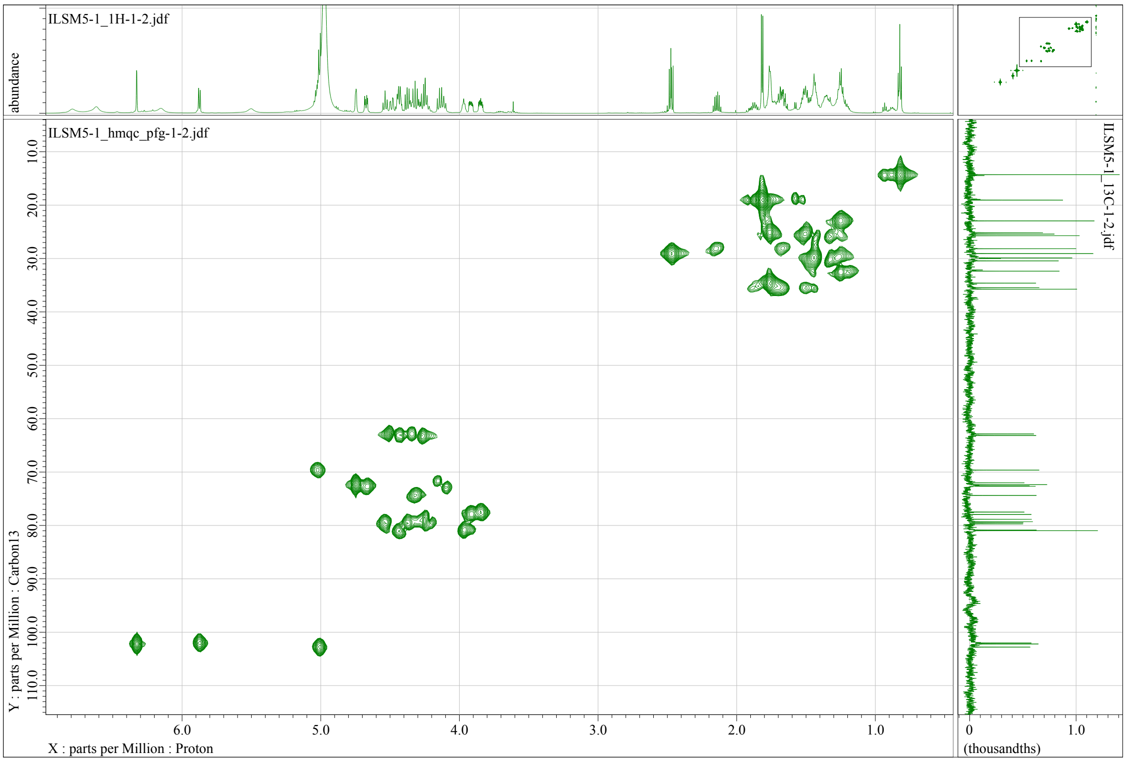

Figure S50: HMQC (600 MHz, pyridine-d_5) spectrum of **5**

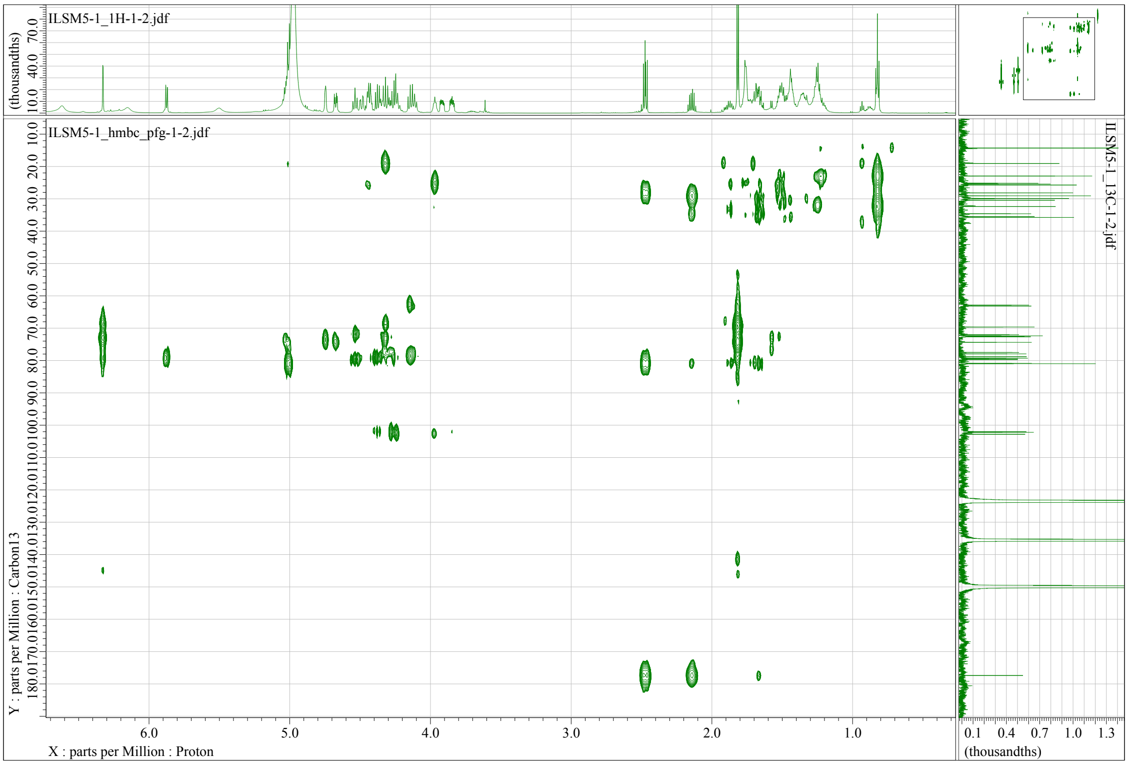

Figure S51: HMBC (600 MHz, pyridine-d_5) spectrum of **5**

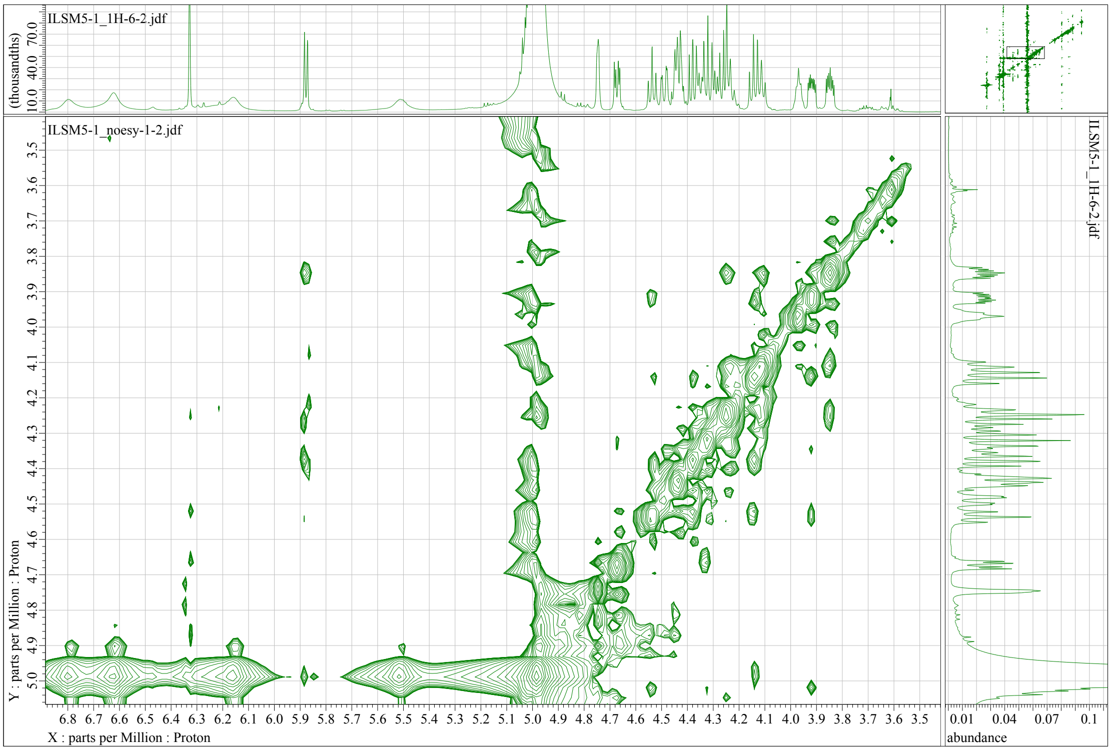

Figure S52: NOESY (600 MHz, pyridine-d_5) spectrum of **5**
